# β-Carboline (FG-7142) modulates fear but not anxiety-like behaviour in zebrafish

**DOI:** 10.1038/s41598-023-51072-6

**Published:** 2024-01-05

**Authors:** Kale R. Scatterty, Trevor J. Hamilton

**Affiliations:** 1 Department of Psychology, MacEwan University, Edmonton, AB T5J 4S2 Canada; 2 Neuroscience and Mental Health Institute, University of Alberta, Edmonton, AB T6G 2R3 Canada

**Keywords:** Emotion, Stress and resilience, Neurochemistry, Neuroscience, Psychology

## Abstract

The β-Carboline FG-7142 is a partial inverse agonist at the benzodiazepine allosteric site on the GABA-A receptor that induces anxiogenic, proconvulsant, and appetite-reducing effects in many species, including humans. Seizure-kindling effects have been well studied, however anxiogenic properties are relatively unexplored. This study aimed to investigate concentration-dependent effects of FG-7142 on anxiety-like behaviour and fear responses in zebrafish (*Danio rerio*) using the open-field test (OF) and novel object approach test (NOA). A U-shaped distribution was found with maximal responses in increased immobility and reduced distance moved at 10 µM in the NOA but not the OF. Follow up experiments demonstrated a lack of effect in repeated OF testing and no changes in opercular movements. Furthermore, the effect of FG-7142 was reversed with ethanol treatment. These results suggest that FG-7142 elicits a ‘freezing’ response in zebrafish via the introduction of novelty, suggesting fear-induction. These findings indicate that FG-7142 may act as an agent to promote acute fear responses in zebrafish.

## Introduction

The β-Carboline FG-7142 is a bioactive indole alkaloid with anorectic (appetite-reducing), proconvulsant (seizure-inducing), and anxiogenic (anxiety-increasing) properties^1–6^. It is a partial inverse agonist at the benzodiazepine allosteric site of the γ-Aminobutyric acid (GABA)-A receptor^3,4,6^. FG-7142 activity at GABA-A receptors decreases GABA transmission (the opposite action of benzodiazepines) resulting in excitatory post-synaptic potential summation and higher-than-normal activation throughout pathways modulated by these receptors^6,7^. This heightened nervous system excitation causes increased arousal, heightened stress, and symptoms associated with mood and anxiety disorders^6,7^ FG-7142 is found in succulent species^8^, hallucinogenic plants^8^, and via combustion of *Nicotiana tabacum* (tobacco) leaves^4^. Elevated FG-7142 levels have been detected via urinalysis in tobacco smokers and in non-smokers at lower levels, suggesting endogenous production^4,9^ and raising questions about what role FG-7142 may have in nervous system excitation and anxiety-related behaviours.

Anxiety and anxiety disorders are pervasive among humans^10,11^, with related fear and trauma disorders being additionally prominent^11,12^. The number of individuals meeting DSM-5 criteria for disorder^13^ is often underreported and true rates are likely higher. Research has thus focused on refining our understanding of how humans experience, react to, learn, habituate to, extinguish, and adapt to anxiety and fear-related symptoms^14–16^, with pharmacological studies on anxiogenic compounds that may heighten or evoke such symptoms, such as FG-7142, often at the forefront of study.

In early research with FG-7142, human participants reported symptoms indicating extreme anxiety, with some requesting emergency benzodiazepine rescue^4^. FG-7142 studies in humans were thus short-lived due to side effect severity^4,5,17^ and focus shifted to non-human mammals for further research^3,4,6^. Early rodent research replicated proconvulsant effects found in humans, finding seizure activity at higher doses and comparable anxiety-like behaviours via suppressed aggression and increased defensive responses^4,6,18,19^. While FG-7142 has well-established seizure-kindling effects^4,6,20^, its anxiogenic properties have garnered recent interest, particularly among researchers using animal models of anxiety-like behaviour^2,4,21^.

Wild-type zebrafish (*Danio rerio*) are an emerging and well-established model organism of anxiety-like behaviours^22–27^ and demonstrate behavioural phenotypic responses indicative of anxiety paralleling humans^23,28–30^. The zebrafish genome is ~ 70% homologous to humans and both species share anatomical and physiological similarities in sensory perception and neurotransmission^31,32^, making such behaviours generalizable. Zebrafish demonstrate responses indicative of anxiety, fear, stress, social behaviours, aggression, aversion, avoidance, and many types of memory on comparable rodent paradigms relevant to humans^25,26,33^. Behavioural markers for anxiety and fear include increased locomotion or avoidance responses in place preference^25,26^. Kalueff and collegues^25^ also note a ‘freezing’ response, characterized by reduced locomotion without reduction in opercular (respiratory/gill) movements, distinct from sedation or immobility and indicative of fear or alarm. These markers allow observable quantification of anxiety-like behaviours with minimal invasiveness, experimental control, and internal validity. Zebrafish are preferred for observing these behaviours over mammals due to their rapid breeding, short gestation period, quick maturation, ease of handling, and high housing density, allowing statistically robust sample sizes^23,28–30^. To date, most research on anxiety in zebrafish has focused on the effects of anxiolytic (anxiety-reducing) drugs (e.g., chlordiazepoxide or diazepam^34^) in zebrafish via paradigms adapted from rodent studies^26,27,35^, but it is also important to investigate compounds that elicit, mediate, or condition fear and anxiety-like behaviours, such as FG-7142, to better understand their mechanisms in the zebrafish model.

In this study we aimed to (1) establish a concentration–response curve and effective concentration (EC) of FG-7142 on zebrafish for fear and/or anxiety like behaviour using an open field (OF) followed by novel object approach (NOA) test; (2) validate this EC by comparing effects with or without a stimulus or drug that modulates any elicited anxiety or fear, such as ethanol; and (3) evaluate the opercular movements of fish immersed in FG-7142. Should zebrafish be shown as an effective model organism of FG-7142-elicited anxiety and/or fear, such a model could be useful in furthering our understanding of the endogenous mechanisms of anxiety, fear, and trauma disorders as well as in research on anxiolytic drug screening for treatment of their symptoms.

## Results

### Open field test

#### Open field test—Locomotion

**Figure 1:**
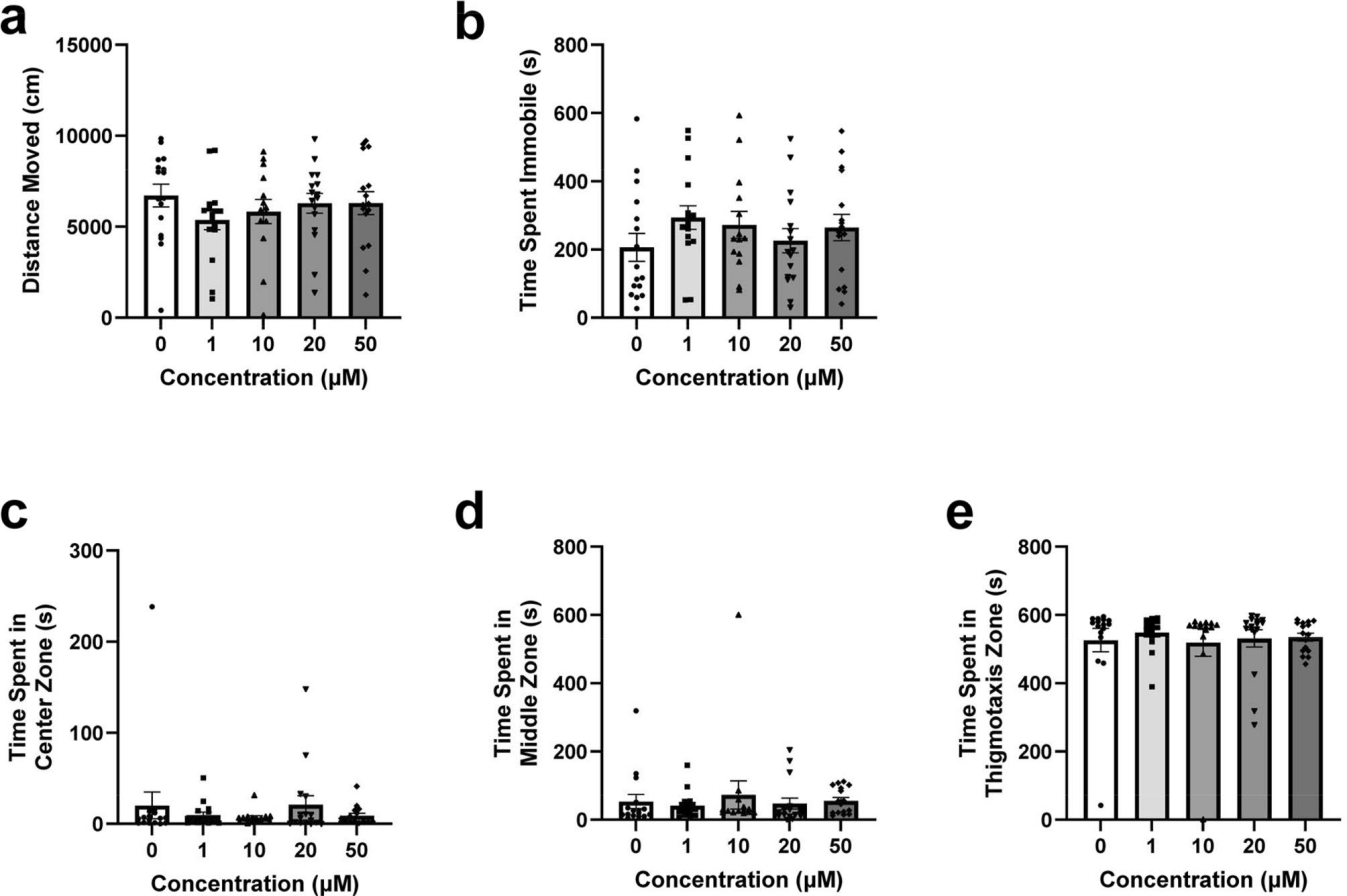
Open field test locomotion & place preference during the OF and NOA tests. Zebrafish locomotor and place preference responses during the OF of the EC-response curve testing. (**a**) Average distance moved by concentration group. (**b**) Average time spent immobile by concentration group. (**c**) Average time spent in the center zone by concentration group. (**d**) Average time spent in the middle zone by concentration group. (**e**) Average time spent in the thigmotaxis zone by concentration group. Error bars represent S.E.M.

All groups across OF locomotor variables except high mobility were normally distributed and the Brown-Forsythe one-way ANOVA model was considered an appropriate fit. A Kruskal–Wallis test was used for high mobility. No significant differences were observed between concentration groups for distance moved (Fig. 1a, *F*(4, 70.99) = 0.7449,* p* = 0.5646) or immobility (Fig. 1b *F*(4, 71.78) = 0.8962, *p* = 0.4709) and no post-hoc comparisons were considered necessary. Post hoc results of mobility and high mobility were similarly not significant and can be seen in the supplemental materials. Overall, zebrafish did not significantly differ in locomotion across concentration groups in the OF.

#### Open field test—Place preference

OF place preference variables were not normally distributed and the Kruskal–Wallis test was considered an appropriate fit. No significant differences were observed between concentration groups for time spent in the center zone (Fig. 1c, *H*(4) = 0.3221, *p* = 0.9883), middle zone (Fig. 1d, *H*(4) = 3.992, *p* = 0.4071), or thigmotaxis zone (Fig. 1e, *H*(4) = 2.717, *p* = 0.6063). No post-hoc comparisons were considered necessary. Overall, zebrafish did not significantly differ in place preference across all concentration groups in the OF.

### Novel object approach test

#### Novel object approach test—Locomotion

**Figure 2:**
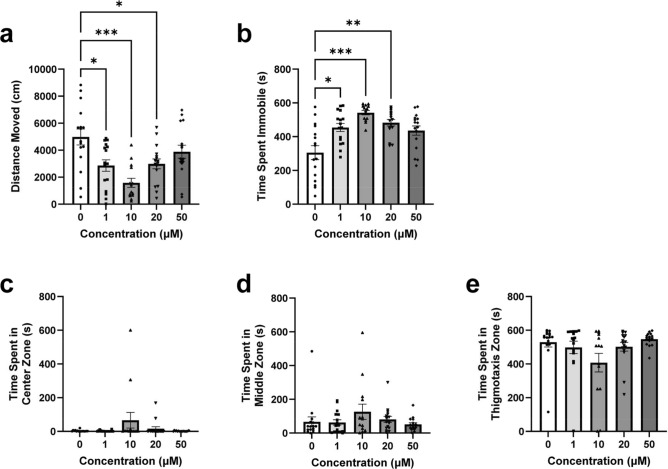
Novel object approach test locomotion & place preference during the OF and NOA tests. Zebrafish locomotor and place preference responses during the NOA of the EC-response curve testing. (**a**) Average distance moved by concentration group. (**b**) Average time spent immobile by concentration group. (**c**) Average time spent in the center zone by concentration group. (**d**) Average time spent in the middle zone by concentration group. (**e**) Average time spent in the thigmotaxis zone by concentration group. Error bars represent S.E.M. *p < 0.05, **p < 0.01, ***p < 0.001.

All groups across the NOA locomotor variables were normally distributed except high mobility and the Brown-Forsythe one-way ANOVA model was considered an appropriate fit. A Kruskal–Wallis test was used for high mobility. A significant difference was found between concentration groups during the NOA for distance moved (Fig. 2a, *F*(4, 62.52) = 7.666, *p* < 0.0001) and Dunnett’s T3 multiple comparisons found significant differences between the 1 µM (*µ* = 2865 cm, *SEM* = 423.4, *p* = 0.0283), 10 µM (*µ* = 1576 cm, *SEM* = 340.0, *p* = 0.0002), and 20 µM (*µ* = 2989 cm, *SEM* = 365.0, *p* = 0.0326) fish vs. the 0 µM control group (*µ* = 4981 cm, *SEM* = 593.3).

A significant difference was also found between the concentration groups during the NOA for immobility (Fig. 2b, F(4, 47.97) = 10.42, *p* < 0.0001) and Dunnett’s T3 multiple comparisons found significant differences between the 1 µM (*µ* = 454.2 s, *SEM* = 96.68, *p* = 0.0181), 10 µM (*µ* = 542.4 s, *SEM* = 12.91, *p* = 0.0001), and 20 µM (*µ* = 482.7 s, *SEM* = 18.97, *p* = 0.0031) fish vs. the 0 µM control group (*µ* = 305.4 s, *SEM* = 41.07).

Lastly, post hoc results of mobility and high mobility during the NOA were similarly significant and can be seen in the supplemental materials. Overall, zebrafish locomotion for the 1 µM, 10 µM, and 20 µM concentration groups significantly decreased across all locomotor variables during the NOA compared to controls, with peak responses found in the 10 µM fish.

#### Novel object approach test—Place preference

NOA place preference variables were not normally distributed, and the Kruskal–Wallis test was considered an appropriate fit. No significant differences were observed between concentration groups for time spent in the center zone (Fig. 2c, *H*(4) = 0.1.346, *p* = 0.8535), middle zone (Fig. 2d, H(4) = 3.143, *p* = 0.5343), or thigmotaxis zone (Fig. 2e, *H*(4) = 5.563, *p* = 0.2342). No post-hoc comparisons were considered necessary. Overall, zebrafish did not significantly differ in place preference across all concentration groups in the NOA.

#### OF-NOA post hoc multifactor analysis

Due to significant differences observed in locomotor variables during the NOA, a post-hoc repeated measures mixed model three-way ANOVA was conducted on the locomotor data. Residual analysis confirmed that assumptions of normality, homogeneity, multicollinearity, and homoscedasticity were met, and it was concluded a repeated measures three-way ANOVA model was a good fit. Due to sphericity violations, a Greenhouse–Geisser correction was implemented to correct p-values.

**Figure 3:**
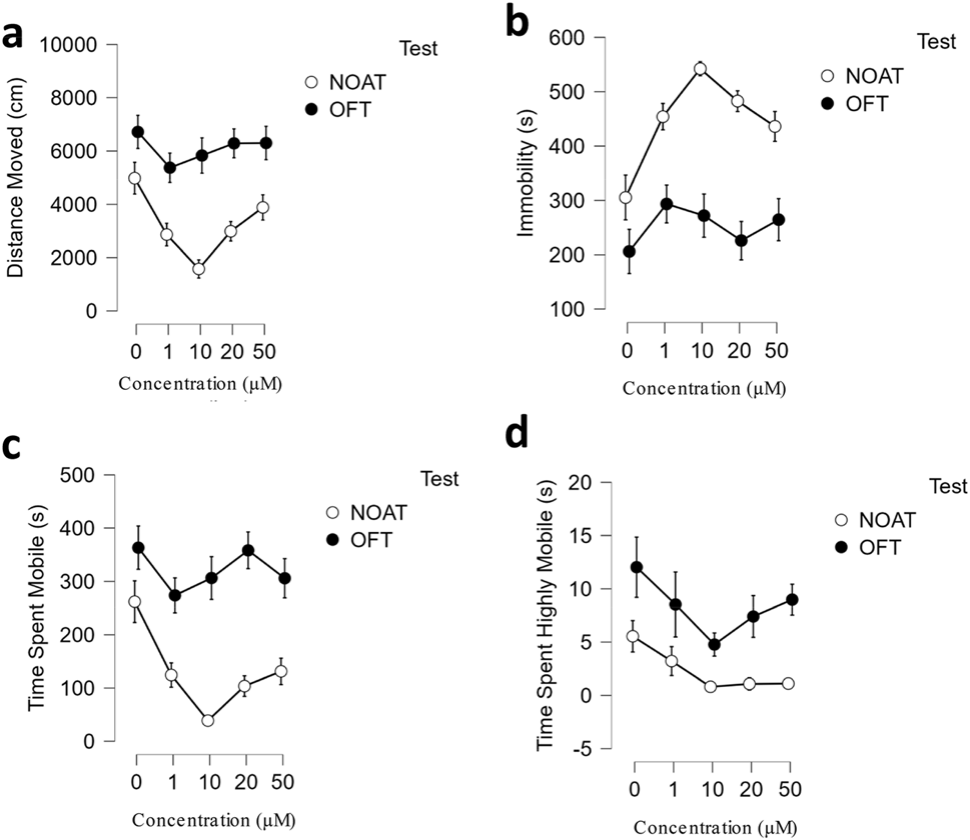
Open field test to novel object approach test post-hoc multifactor analysis. Evaluation of main and interaction effects between locomotor variables of the EC-response curve testing. (**a**) Average distance moved by concentration group and test. (**b**) Average time spent immobile by concentration group and test. (**c**) Average time spent mobile by concentration group and test. (**d**) Average time spent highly mobile by concentration group and test. Error bars represent S.E.M. Lines between points represent trendlines between concentrations.

Significant main effects were found for OF-NOA locomotion (*F*(1.002) = 704.577, *p* < 0.001, *η*^*2*^ = 0.686), concentration (*F*(4) = 4.842, *p* = 0.001, *η*^*2*^ = 0.006), and test (*F*(1) = 71.435, *p* < 0.001, *η*^*2*^ = 0.023). Significant two-factor interaction effects were observed for locomotion* concentration (*F*(4.008) = 4.879, *p* < 0.001, *η*^*2*^ = 0.019) and locomotion*test (*F*(1.002) = 71.229, *p* = 0.001, *η*^*2*^ = 0.069), but not for concentration *test (*F*(4) = 1.563, *p* = 0.187, *η*^*2*^ = 0.002). Lastly, no significant three-way interaction was observed for locomotion*concentration*test (*F*(4.008) = 1.578, *p* = 0.183, *η*^*2*^ = 0.006). In summary, a large but expected main effect was found for locomotion on locomotor variables while concentration and test had small and small-to-moderate main effects, respectively, and locomotion had a small effect that was dependent on the concentration exposure received on locomotor variables and a moderate effect that was dependent on the test (Table 1, Fig. 3). A post-hoc comparison was conducted using a repeated measures two-way ANOVA to evaluate group-specific changes in place preference for the center zone as a measure of object approach and no groups demonstrated any group-specific changes in preference for or away from the center zone between the OF and NOA. These post-hoc results can be found in the supplemental materials.

**Table 1:** Open Field Test to Novel Object Approach Test Post-Hoc Multifactor Analysis.

Cases	Sphericity Correction	Sum of Squares	df	Mean Square	F	p	η^2^
Within subjects effects							
Locomotion	Greenhouse–Geisser	2.359 × 10^+9^	1.002	2. 354 × 10^+9^	704.577	< .001	0.686
Locomotion ✻ Concentration	Greenhouse–Geisser	6.534 × 10^+7^	4.008	1.630 × 10^+7^	4. 879	< .001	0.019
Locomotion ✻ Test	Greenhouse–Geisser	2.384 × 10^+8^	1.002	2.380 × 10^+8^	71. 229	0.001	0.069
Locomotion ✻ Concentration ✻ Test	Greenhouse–Geisser	2.113 × 10^+7^	4.008	5.272 × 10^+6^	1. 578	0.183	0.006
Residuals	Greenhouse–Geisser	4.887 × 10^+8^	146.280	3.341 × 10^+6^			

Cases	Sum of squares	df	Mean Square	F	p	η^2^	
Between subjects effects							
Concentration	2.137 × 10^+7^	4	5.342 × 10^+6^	4.842	0.001	0.006	
Test	7.882 × 10^+7^	1	7.882 × 10^+7^	71.435	< .001	0.023	
Concentration ✻ Test	6.897 × 10^+6^	4	1.742 × 10^+6^	1.563	0.187	0.002	
Residuals	1.611 × 10^+8^	146	1.103 × 10^+6^				

Note: Type III Sum of Squares.

### Repeated open field test

#### First open field test

All groups were normally distributed on the variable of distance moved and Welch’s t-test was considered an appropriate fit. Immobility was not found to be normally distributed so a Mann–Whitney test was used. No significant differences were observed between concentration groups for distance moved (Supp. Figure 2a, *t*(26.43) = 1.524, *p* = 0.1395). A significant difference was, however, found between concentration groups for immobility (Supp. Figure 2b, *U* = 68, *p* = 0.0234) in which 10 µM fish (*Mdn* = 65.40 s) spent significantly more time immobile than 0 µM fish (*Mdn* = 14.00 s). Post hoc results of mobility and high mobility were similarly significant and can be seen in the supplemental materials.

None of the OF place preference variables were normally distributed and the Mann–Whitney test was considered an appropriate fit. No significant differences were observed between concentration groups for time spent in the thigmotaxis zone (Supp. Figure 2c, *U* = 99, *p* = 0.2871) or middle zone (Supp. Figure 2d, *U* = 111, *p* = 0.5391), however, a significant difference was found for time spent in the center zone (Supp. Figure 2e, *U* = 69, *p* = 0.0260) in which 10 µM (*Mdn* = 37.96 s) spent significantly more time in the center zone than 0 µM fish (*Mdn* = 13.20).

Overall, 10 µM zebrafish differed from the 0 µM fish only in that they spent significantly more time immobile and significantly more time in the center zone during the first OF. These results can be seen graphically in Fig. 2 of the supplemental materials.

#### Second open field test

**Figure 4:**
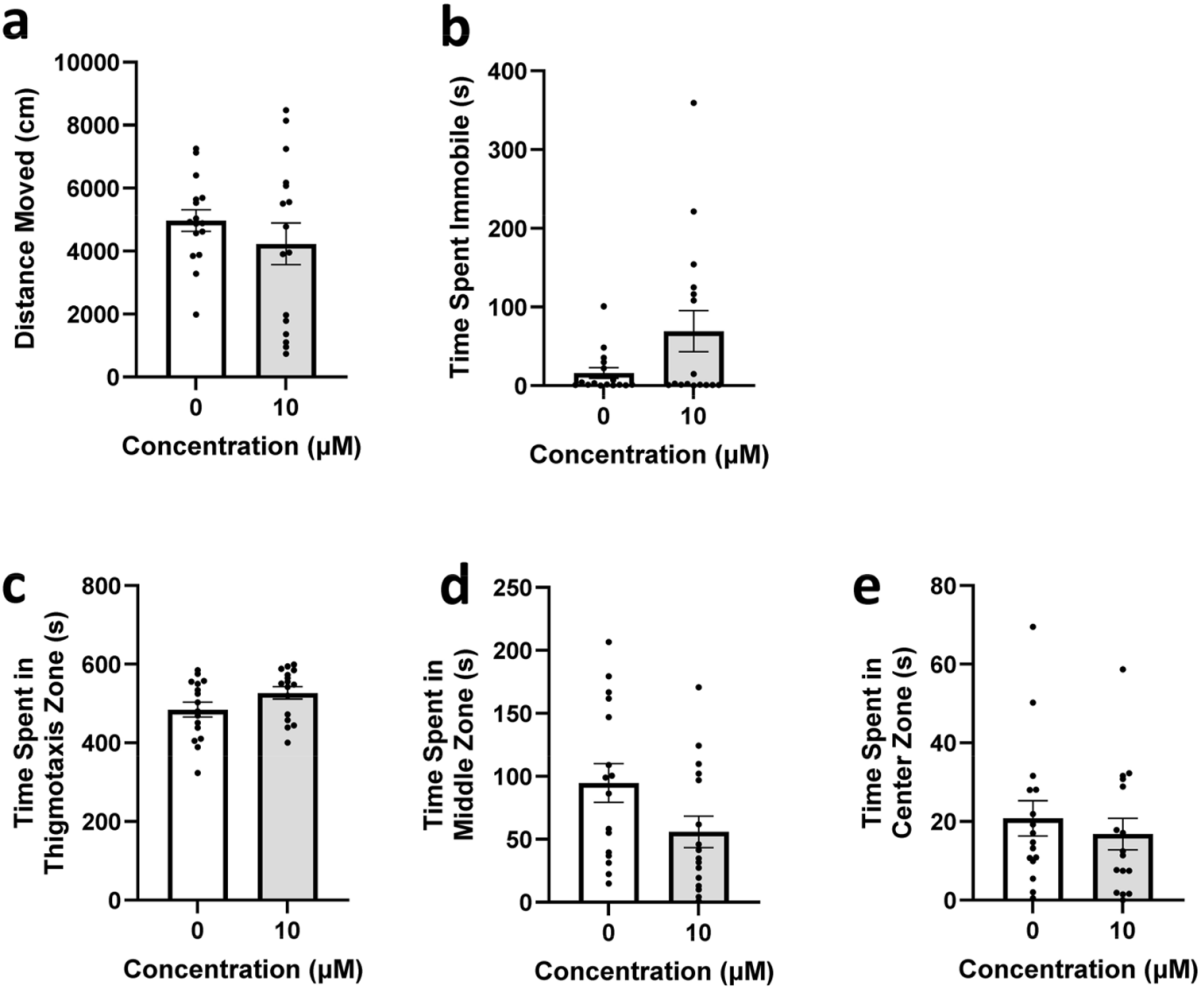
Second open field test locomotion and place preference during the open field test to open field test procedure. Zebrafish locomotor and place preference responses during the OF-OF testing. (**a**) Average distance moved by concentration group. (**b**) Average time spent immobile by concentration group. (**c**) Average time spent in the thigmotaxis zone by concentration group. (**d**) Average time spent in the middle zone by concentration group. (**e**) Average time spent in the center zone by concentration group. Error bars represent S.E.M.

All groups were normally distributed on the variable of distance moved and Welch’s t-test was considered an appropriate fit. Immobility was not found to be normally distributed, and a Mann–Whitney test was used instead. No significant differences were observed between concentration groups for distance moved (Fig. 4a, *t*(22.39) = 0.9924, *p* = 0.3316) or immobility (Fig. 4b, U = 104, *p* = 0.3756). Post hoc results of mobility and high mobility similarly had no significant differences and can be seen in the supplemental materials.

Distributions for time spent in the middle and thigmotaxis zones were normal and Welch’s test was considered an appropriate fit. Time spent in the center zone was not normally distributed and a Mann-Whintey test was used instead. No significant differences were observed between concentration groups for time spent in the thigmotaxis zone (Fig. 4c, *t*(29.05) = 1.718, *p* = 0.0965), middle zone (Fig. 4d, *t*(28.77) = 1.946, *p* = 0.0614), or center zone (Fig. 4e, (*U* = 110.5, *p* = 0.5207).

Overall, no significant differences were observed for either locomotor or place preference variables during the second OF.

### Ethanol challenge

#### Ethanol—Open field test

**Figure 5:**
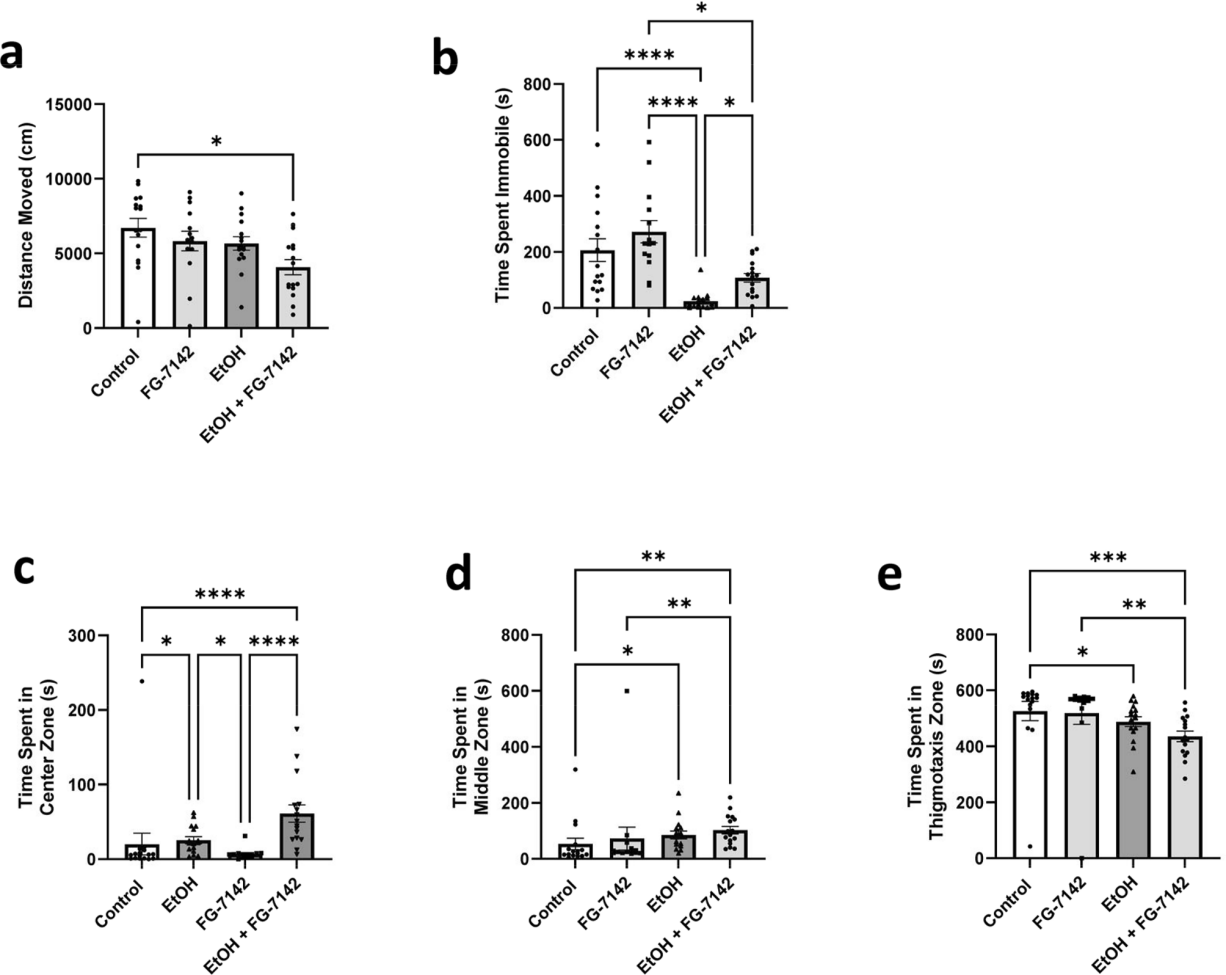
Open field test locomotion & place preference during with EtOH challenge. Zebrafish locomotoion and place preference responses during the comparison of FG-7142 with or without the presence of EtOH. (**a**) Average distance moved by concentration group. (**b**) Average time spent immobile by concentration group. (**c**) Average time spent in the center zone by concentration group. (**d**) Average time spent in the middle zone by concentration group. (**e**) Average time spent in the thigmotaxis zone by concentration group. *p < 0.05, **p < 0.01, ***p < 0.001, ****p < 0.0001.

In all groups, distance moved was normally distributed and the Brown-Forsythe one-way ANOVA model was considered an appropriate fit. Immobility was not normally distributed, and a Kruskal–Wallis test was used. A significant difference was found between groups in distance moved (Fig. 5a, F(3, 52.77) = 3.892, *p* = 0.0138) and Dunnett’s T3 multiple comparisons found that fish in the EtOH + FG-7142 group (*µ* = 4077 cm, *SEM* = 508.5) moved significantly less (*t*(28.86) = 3.288, *p* = 0.0155) than controls (*µ* = 6718 cm, *SEM* = 622.2). A significant difference was also found between groups in immobility (Fig. 5b, H(4, 62) = 35.38, *p* < 0.0001) and Dunn’s multiple comparisons found that the EtOH group (*M* = 10.81, *SEM* = 8.324) spent significantly less time immobile than the FG-7142 group (*M* = 47.64, *SEM* = 39.77;* z* = 5.578, *p* < 0.0001), EtOH + FG-7142 group (*M* = 30.19, *SEM* = 15.74;* z* = 3.038, *p* = 0.0143), and controls (*M* = 39.38, *SEM* = 40.60;* z* = 4.478, *p* < 0.0001), while the FG-7142 group (*M* = 47.64, *SEM* = 39.77) was found to spend significantly more time immobile than the EtOH + FG-7142 group (*M* = 30.19, *SEM* = 15.74; *z* = 2.644, *p* = 0.0492). Results for mobility and high mobility were similarly significant and can be found in the supplemental materials.

Regarding overall locomotion, zebrafish in the EtOH + FG-7142 group moved a significantly shorter distance than controls; the EtOH group spent significantly less time immobile than all groups while the FG-7142 group spent significantly more time immobile than controls and EtOH + FG-7142 fish, and the EtOH + FG-7142 group spent significantly less time immobile than controls.

All OF place preference variables were not normally distributed and the Kruskal–Wallis test was considered an appropriate fit. A significant difference was found between groups for time spent in the center zone (Fig. 5c, *H*(4, 62) = 30.21, *p* < 0.0001) and Dunn’s multiple comparisons showed EtOH fish (*M* = 36.56, *SEM* = 4.631) spent significantly more time in the center zone than the FG-7142 (*M* = 19.07, *SEM* = 1.964; *z* = 2.649, *p* = 0.0484) and controls (*M* = 19.63, *SEM* = 14.60; *z* = 2.655, *p* = 0.0475) while FG-7142 fish (*M* = 19.07, *SEM* = 1.964) also spent significantly less time in the center zone than the EtOH + FG-7142 group (*M* = 49.19, *SEM* = 11.62; *z* = 4.561, *p* < 0.0001) and the EtOH + FG-7142 group (*M* = 49.19, *SEM* = 11.62) spent significantly more time in the center zone than controls (*M* = 19.63, *SEM* = 14.60; *z* = 4.635, *p* < 0.0001). A significant difference was found between groups for time spent in the middle zone (Fig. 5d, *H*(4, 62) = 19.81, *p* = 0.0002) and Dunn’s multiple comparisons showed that EtOH + FG-7142 fish (*M* = 43.69, *SEM* = 13.74) spent significantly more time in the middle zone than the FG-7142 group (*M* = 22.43, *SEM* = 40.83; *z* = 3.220, *p* = 0.0077) and controls (*M* = 20.06, *SEM* = 20.16; *z* = 3.704, *p* = 0.0013) while EtOH fish (*M* = 38.69, *SEM* = 13.78) spent significantly more time in the middle zone than controls (*M* = 20.06, *SEM* = 20.16; *z* = 2.920, *p* = 0.0210). Lastly, a significant difference was found between groups for time spent in the thigmotaxis zone (Fig. 5e, *H*(4, 62) = 23.95, *p* < 0.0001) and Dunn’s multiple comparisons found that the EtOH + FG-7142 group (*M* = 16.56, *SEM* = 18.53) spent significantly less time in the thigmotaxis zone than the FG-7142 group (*M* = 41.00, *SEM* = 40.47; *z* = 3.701, *p* = 0.0013) and controls (*M* = 43.84, *SEM* = 33.86; *z* = 4.277, *p* = 0.0001) while the EtOH group (*M* = 25.78, *SEM* = 17.68) spent significantly less time in the thigmotaxis zone than controls (*M* = 43.84, *SEM* = 33.86; *z* = 2.832, *p* = 0.0278).

Regarding overall location preference, EtOH, as well as the presence of EtOH with FG-7142, showed distinct effects that were significantly different to the effects of FG-7142 alone. The combined presence of EtOH and FG-7142 was significantly different from FG-7142 on all place preference variables in the OF.

#### Ethanol—Novel object approach test

**Figure 6:**
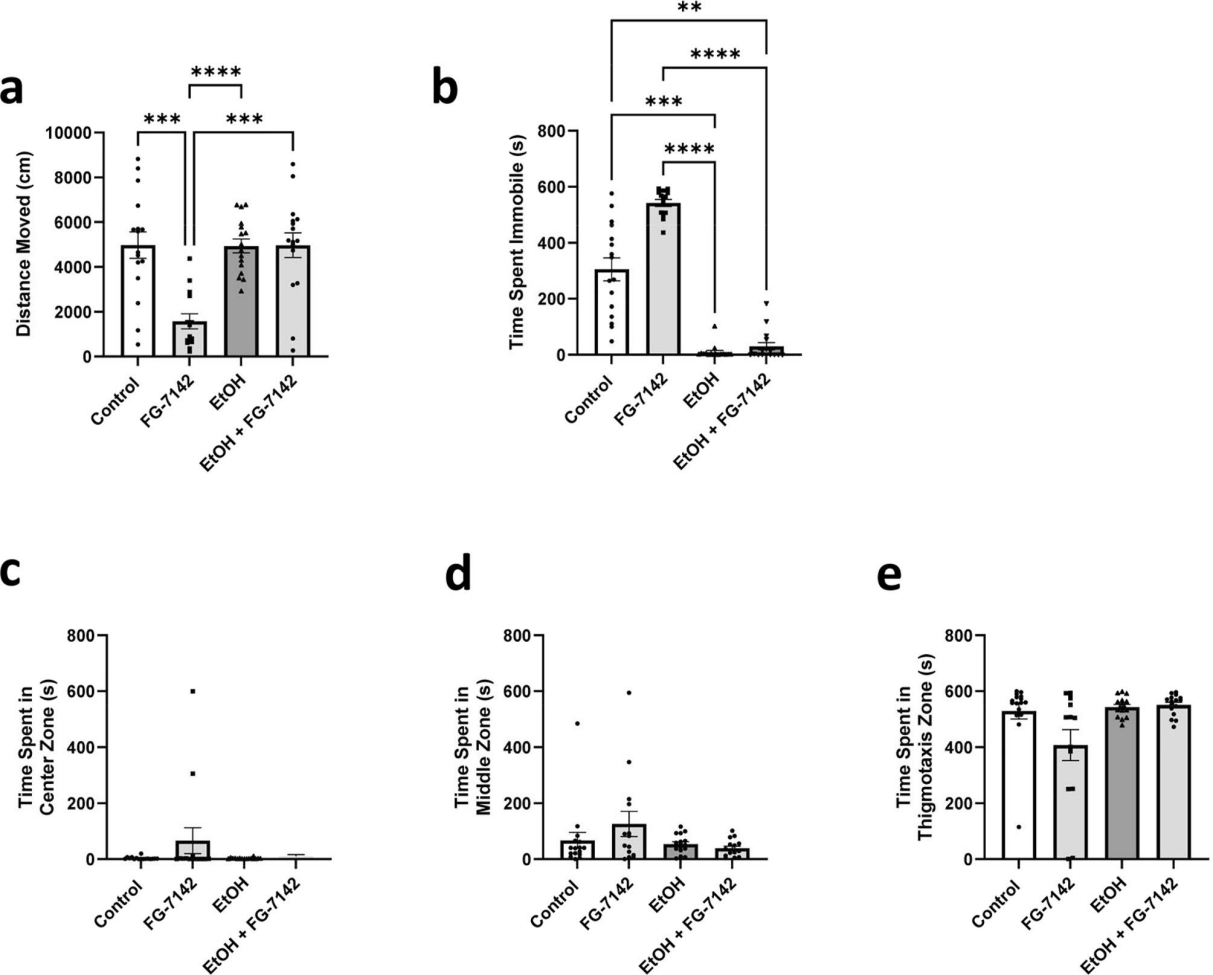
Novel object approach test locomotion & place preference with EtOH challenge. Zebrafish locomotoion and place preference responses during the comparison of FG-7142 with or without the presence of EtOH. (**a**) Average distance moved by concentration group. (**b**) Average time spent immobile by concentration group. (**c**) Average time spent in the center zone by concentration group. (**d**) Average time spent in the middle zone by concentration group. (**e**) Average time spent in the thigmotaxis zone by concentration group. **p < 0.01, ***p < 0.001, ****p < 0.0001.

For all groups distance moved was normally distributed, and the Brown-Forsythe one-way ANOVA model was considered an appropriate fit. Immobility was not normally distributed, and a Kruskal–Wallis test was used instead. A significant difference was found between groups in distance moved (Fig. 6a, F(3, 46.32) = 12.14, *p* < 0.0001) and Dunnett’s T3 multiple comparisons found that fish in the FG-7142 group (*µ* = 1576 cm, *SEM* = 340.0) moved significantly less than EtOH fish (*µ* = 4940 cm, *SEM* = 312.1; *t*(27.32) = 7.288, *p* < 0.0001), EtOH + FG-7142 fish (*µ* = 4975 cm, *SEM* = 555.9; *t*(24.38) = 5.215, *p* = 0.0001), and controls (*µ* = 4981 cm, *SEM* = 593.3; *t*(23.54) = 4.979 *p* = 0.0003). A significant difference was also found between groups in immobility (Fig. 6b, H(4, 62) = 47.83, *p* < 0.0001) and Dunn’s multiple comparisons found that the FG-7142 group (*M* = 54.36, *SEM* = 12.91) spent significantly more time immobile than the EtOH group (*M* = 15.75, *SEM* = 6.268;* z* = 5.847, *p* < 0.0001) and EtOH + FG-7142 group (*M* = 18.00, *SEM* = 13.20;* z* = 5.507, *p* < 0.0001), while the controls (*M* = 40.75, *SEM* = 41.07) also spent significantly more time immobile than the EtOH fish (*M* = 15.75, *SEM* = 6.268; *z* = 3.919, *p* = 0.0005) and EtOH + FG-7142 fish (*M* = 18.00, *SEM* = 13.20; *z* = 3.567, *p* = 0.0022). Post hoc results for time spent mobile and highly mobile were similarly significant and can be found in the supplemental materials.

Regarding overall locomotion, the FG-7142 group moved a significantly shorter distance than all other groups and the FG-7142 group spent significantly more time immobile than the EtOH and EtOH + FG-7142 groups while both groups spent significantly less time immobile than controls.

For all groups, NOA place preference variables were not normally distributed, and the Kruskal–Wallis test was considered an appropriate fit. No significant difference was found between groups for time spent in the center zone (Fig. 6c, *H*(4, 62) = 0.7719, *p* = 0.8562) and no post-hoc multiple comparisons were considered necessary. No significant difference was found between groups for time spent in the middle zone (Fig. 6d, *H*(4, 62) = 2.991, *p* = 0.3930) and no post-hoc multiple comparisons were considered necessary. Lastly, no significant difference was found between groups for time spent in the thigmotaxis zone (Fig. 6e, *H*(4, 62) = 5.222, *p* = 0.1563) and no post-hoc multiple comparisons were considered necessary.

Regarding overall location preference, no evidence was found of any significant differences between any of the groups on time spent in any arena zone during the NOA.

### Opercular movements

Residual analysis confirmed assumptions of normality, homogeneity, multicollinearity, and homoscedasticity were met and a repeated measures two-way ANOVA model was considered a good fit for the opercular movement data.

No significant main effects were found for concentration group (*F*(2, 135) = 0.8932, *p* = 0.4117) or time (*F*(4, 135) = 0.6510, *p* = 0.6271) and no significant interaction effects were found between concentration group and time (*F*(8, 135) = 0.1450, *p* = 0.9968). Because no significant main or interaction effects were found or borderline, no post-hoc multiple comparisons were deemed necessary.

**Figure 7:**
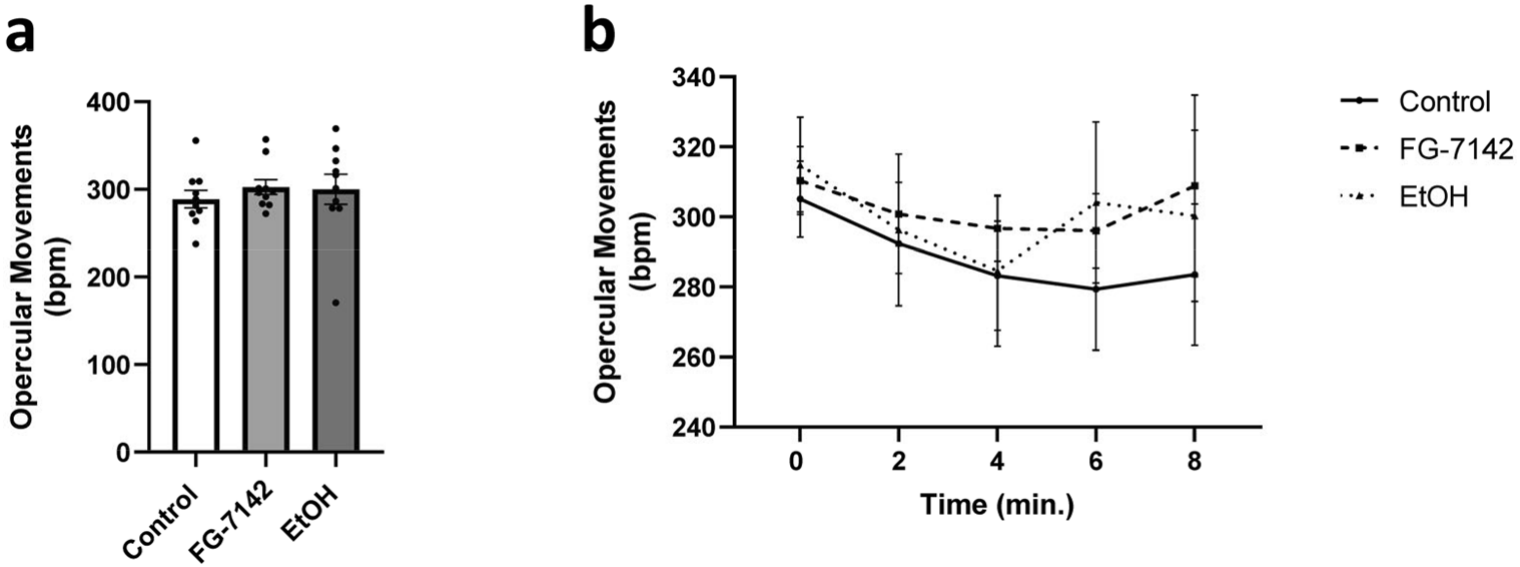
Opercular movement testing. Zebrafish opercular BPM while in habitat water, habitat water and FG-7142, or habitat water and ethanol. (**a**) Average opercular BPM by concentration group. (**b**) Average opercular BPM by concentration group and time in 2-min intervals. Error bars represent S.E.M. Lines between points represent trendlines over time between concentration group mean BPMs.

Overall, fish did not appear to differ significantly in opercular movements over time between groups (Fig. 7a), nor were any significant effects of concentration group or time discovered (Fig. 7b).

## Discussion

This study sought to investigate the effect of FG-7142 on zebrafish fear and anxiety-like behaviour. A concentration–response curve was successfully established with a U-shaped distribution and EC of 10 µM. Further experimentation showed that effects were stimulus-dependent, reduced via addition of ethanol, and elicit behavioural phenotypes of freezing via nondepressed opercular movements. Together, these findings suggest that FG-7142 elicited fear and not anxiety-like responses in zebrafish.

We found consistent decreases in locomotion in the NOA when the novel stimulus was added to the arena, but not OF with no stimulus present, with peak responses occurring in the NOA at 10 µM. This suggests that FG-7142 heightens the fear response to an unfamiliar object. Noteworthy is the lack of changes in proxies of anxiety-like behaviour (i.e. thigmotaxis) in either the OF or NOA with any concentration of FG-7142. A U-shaped distribution of concentration-dependent toxicity (Hormesis) was found, consistent with some existing literature^36^. In other words, as the concentration increased toward 10 µM, effects on locomotion increased, while as the concentration increased past 10 µM, these effects decreased. Hormetic data are not highly susceptible to false-positive rates and occur more frequently than often expected^37,38^. While the cause is unique to each compound, interaction effects have been implicated in some hormetic curves^37,39^, as was found between concentration and test (OF vs. NOA). Hormesis could also be explained by homeostatic compensatory responses that may offset the effects of FG-7142 at a certain threshold^37,39^, which could be likely given its endogenous production^4^ (although endogenous production is unknown in fish). Future studies could evaluate other neurotransmitters or stress-related hormones for changes that may explain the physiological basis of FG-7142 hormesis in zebrafish. Furthermore, cognitive neuroimaging studies–as outlined by Hamilton, Allen, & Reynolds^40^–could monitor brain activity across FG-7142 concentrations and assess whether heightened excitation and arousal parallels behaviourally observed hormesis.

In contrast to previous studies, the EC identified in this study surpassed 0.50 µM reported by Lopez Patino and colleagues^36^ and 2.5 µM by Steenbergen and colleagues^41^. The FG-7142 concentration (group) had an independent small effect as well as a small effect that was dependent upon the test condition (OF vs. NOA), while the test condition yielded a moderate effect on locomotion. These variations may stem from the use of different experimental paradigms; Lopez Patino and colleagues^36^ used high environmental stimulation tanks and Steenbergen and colleagues^41^ used light/dark paradigms. Additionally, Lopez Patino and colleagues^36^ used adult zebrafish with FG-7142 dissolved in ethanol while Steenbergen and colleagues^41^ used juvenile zebrafish with FG-7142 dissolved in DMSO. Future experiments could explore concentrations between 1.0 and 10 µM and using behavioural tests with fear-inducing stimuli such as vibration or “tapping” of the arena walls^42^, alarm substance extracted from deceased or injured zebrafish^43^, or introduction of predator-like stimuli such as sound or physical replicas^44^.

To explore the physiological impact on the cardiovascular system we assessed opercular movements during exposure to FG-7142. Opercular movements were not altered by FG-7142 compared to controls, contrary to sedative or anxiolytic effects seen with drugs like chlordiazepoxide and diazepam^25,26,28^. This observation, combined with significantly reduced locomotion, indicates fear-like freezing behaviour^25^. Freezing was also significant in 1.0 µM and 20 µM fish, merely to a lesser extent than 10 µM fish, showing a toxicity-based trend that is not anomalous. This effect was observed exclusively in the presence of a novel stimulus and could be modified or alleviated with the addition of an anxiolytic. Thus, the second goal of this study was also considered accomplished.

While not as pronounced as in the NOA, there was a non-significant OF trend in locomotor reduction in concentration groups compared to controls. It is possible arousal was heightened in the OF and only exacerbated by an introduced stimulus. This is consistent with traditional arousal theories of emotion in which individuals associate heightened physiological states with fear or alarm and attribute this to a perceived stimulus^45,46^. Alternatively, zebrafish locomotion could have been lowered due to acclimation to the stimulus-free OF over time, as utilized by Stewart and colleagues^27^ as a pre-NOA staging period. Netting the fish into the arena could act as a stimulus that, once removed, allowed fish time to “calm down” following transfer. This could confound any locomotor reductions that may have been significant if fish were otherwise already acclimated. Our repeated OF-OF test where the lack of stimulus (NOA) in the second stage elicited no significant effect, suggests OF fish may be primed via FG-7142 for a fear response prior to stimulus presentation. This also demonstrates that an effect in the NOA and not OF is not due to delayed pharmacological action of FG-7142. Lastly, because thigmotaxis behaviour only assesses horizontal place preference and does not evaluate the same behavioural phenotypes or mechanisms of vertical diving behaviours, other tests may be more sensitive to freezing behaviours, such as the novel tank dive test (NTD)^27,34,47^. Future studies could use a NTD to measure vertical locomotion and confirm whether freezing behaviours are stimulus-dependent or generalized regardless of stimuli, as well as validate these effects by demonstrating whether they elicit both thigmotaxis as well as diving behaviours.

Place preference and place avoidance are established valid measures of increased anxiety^25,26,48,49^, however, anxiety and fear are different states of behaviour. Differentially increased time spent in the thigmotaxis zone in the OF and NOA would have indicated increased anxiety^25^, but this was not observed either between or within groups. During the challenge with ethanol, by changing the behavioural state of the fish the effect of FG-7142 was decreased or fully blocked. Ethanol was chosen because of its reliable effect in zebrafish^20,24–26,50^ but more specific GABA-A receptor agonists could be used to explore the mechanistic effects of FG-7142. Taken together, our study shows a lack of anxiogenic responses induced by FG-7142 and an induction of what we interpret as fear behaviour. Lastly, while recent studies have shown locomotor variables like immobility may not accurately predict anxiety-like responses in zebrafish under the effects of anxiolytic (not anxiogenic) drugs^51^, this concern is not relevant to freezing^25,52^.

To further investigate the fear-inducing effects of FG-7142, altering exposure times may yield differential responses, with longer exposure times potentially eliciting greater effects. This would also validate this model further as a useful tool to investigate fear responses in zebrafish and amenable to conditioned fear paradigms. Future studies could also explore other stimuli, such as the light–dark paradigm, colour preference, predatory or aversive sounds, or mechanical water disturbance to determine whether these fear responses are generalizable.

## Methods

### Animals and handling

Adult wild-type zebrafish (*Danio rerio*) were obtained from MacEwan University’s laboratory breeding facility in August 2022 and October 2022 and were selectively sexed to be ~ 50:50 male:female in each experiment (*n* = 174). These fish were bred at MacEwan University for 13 generations from a wild-type strain originally obtained from the University of Ottawa’s (Ottawa, Ontario, Canada) in-house colony. Gender effects were not explored as previous literature found no significant male–female differences for FG-7142 on zebrafish anxiety^36^. Zebrafish were housed in a ZebTec habitat with four multilinking racks and a centralized water treatment unit (Techniplast, Montreal, QC, Canada) in 10 L tanks for naïve fish with a density of up to 50 fish and 3L tanks for tested fish with a density of up to 15 fish. Husbandry and water quality maintenance were carried out by laboratory technicians. This included regulation of pH between 6.7 and 7.2 (setpoint = 7.1) and temperature between 27 and 29 °C (setpoint = 28.5 °C) throughout the duration of this study, with water recirculated at a 15% per day, purified via carbon filtering and ultraviolet irradiation continuously, and maintained at a conductivity of 1000µS. The habitat room photoperiod alternated on a rotating light–dark cycle with daytime luminance beginning at 7:00 AM and nighttime luminance beginning at 9:00 PM automatically. Fish were fed with fish flakes (Gemma Micro 300, Skretting Ltd, France) or shrimp (Omega One Freeze Dried Shrimp Nutri-Treat, Omega Sea Ltd.) once per day and all experimental testing was conducted prior to daily feeding as outlined by Hamilton and colleagues^49^. Each fish was tested only once to remain naïve to behavioural testing and housed in separate 3L tanks. Fish were observed and visually assessed for signs of distress, neurological and motor impairment, or death. All fish were washed in a separate container of clean habitat water after testing periods before being returned to the recirculated 3L habitat tank. No anesthesia or euthanasia was administered during the duration of this study.

### Drug preparation

FG-7142 was purchased from Sigma-Aldrich Canada Co. (Oakville, ON, Canada) and stored in a dry and dark drug storage cabinet at an ambient room temperature of ~ 23 °C within the key-locked behavioural neuroscience lab at MacEwan University. FG-7142’s carboxamide structure makes it naturally hydrophobic and insoluble in water without an appropriate vehicle^1,8^, thus a stock treatment solution of 14.0 mM FG-7142 dissolved in 10% DMSO as a vehicle was prepared based on a factor of four^36^ or five and a half^41^ of the concentrations used in previous studies with zebrafish. The solution was prepared in a MacEwan university organic chemistry laboratory with appropriate skin and respiratory protection underneath a fume hood. The stock solution was refrigerated at ~ 3.0 °C until experimentation. Because the freezing point of DMSO is 19 °C, each vial was only thawed once to retain compound integrity and minimize the risk of solution separation via oiling-out. Any unused solution was kept in a room temperature (~ 23 °C) dry and dark drug cabinet and to be used within the next testing day. Thawed stock solution that was left unused for more than 5 days was marked and discarded.

### Drug administration

FG-7142 immersion solutions were prepared in a 600 mL beaker prior to testing and maintained within a range of 26.5–28.5 °C via an aquatic heating mat. pH was measured for each experimental treatment and was between 6.9 and 7.5. Each treatment concentration of FG-7142 was added to a beaker of habitat water for a target solution volume of 400 mL and stirred vigorously before immersion. The treatment solution was allowed to stand for 20 min before testing and observed to ensure there was no separation of the compound out of the solution. Final tested FG-7142 immersion concentrations in this study were 0 µM (0.0 µL_*stock*_ + 400 mL_*water*_; *n* = 16 fish), 1.0 µM (28.5 µL_*stock*_ + 399.97 mL_*water*_; *n* = 16 fish), 10 µM (285.0 µL_*stock*_ + 399.71 mL_*water*_; *n* = 16 fish), 20 µM (570.0 µL_*stock*_ + 399.43 mL_*water*_; *n* = 16 fish), and 50 µM (1.425 mL_*stock*_ + 398.58 mL_*water*_; *n* = 16 fish) FG-7142. Although the final concentrations of DMSO varied among the treatment groups, previous findings have established that such concentrations do not significantly impact behaviour^53^.

Each dosing container was surrounded by white corrugated plastic to control for potentially confounding visual stimuli^54^. Naïve fish from each randomly assigned group were netted and placed individually in their respective dosing containers and a clear acrylic cover was placed overtop to avoid fish escaping the beaker. Fish were left in their respective dosing containers for 10 min before being transferred into the subsequent testing arena. Habitat water without FG-7142 present was discarded in the laboratory sink while FG-7142 contaminated water was stored in an appropriate biotoxic waste container.

### Behavioural testing

All experimental testing was conducted during the daylight cycle of the habitat photoperiod between 8:00 AM and 5:00 PM, with ambient luminance in the testing apparatus maintained at ~ 32 cd/m^3^ (cal SPOT photometer; Cooke Corp. CA, USA). All testing arenas, dosing chambers, and habitat tanks were kept on heating pads to maintain a water temperature of 26–28 °C. Arena temperature was measured between each trial and water was changed every 3 trials to control for behavioural confounds and potential cumulative increases in arena water cortisol^55^. Motion tracking of zebrafish behaviour was recorded via a Basler GenICam acA1300-60gc Area Scan video camera (Basler Inc., USA) mounted ~ 1 m above each testing arena. Zebrafish motion captured via this footage was tracked with Noldus EthoVision XT® tracking software (v. 11.0, Noldus, Wageningen, NL) with the differencing settings set to ‘darker’, as the fish would appear dark against the white bottom of the testing arenas. Control fish were interspersed evenly across all testing groups to control for different-day and time-of-day effects that may confound behavioural responses. Researchers were not experimentally blinded to the treatment group of each fish, but all fish were tested under identical conditions and assessed with the same procedure and methods of evaluation and measurement.

#### Open field test and novel object approach test

The OF and NOA are well-validated measures of anxious and aversive phenotypic responses^22,24–27,29,48^. Fear-like or anxiogenic effects were validated via replication using two OFs (vs. OF-NOA) to assess stimulus-dependence, a comparison with-and-without ethanol (EtOH) to investigate mitigation, and measurement of opercular movements to distinguish between freezing and sedative immobility. Increases in both locomotion and thigmotaxis behaviour (wall-hugging) were interpreted as increased anxiety whereas decreased locomotion and nondepressed opercular movements were interpreted as ‘freezing’ behaviour indicating fear. In the OF, these behaviours indicate a reduction in exploratory behaviour as a marker of anxiety and aversion within a novel environment whereas in the NOA, they indicate an increase in avoidance and reduction in approach behaviour to the novel stimulus.

**Figure 8:**
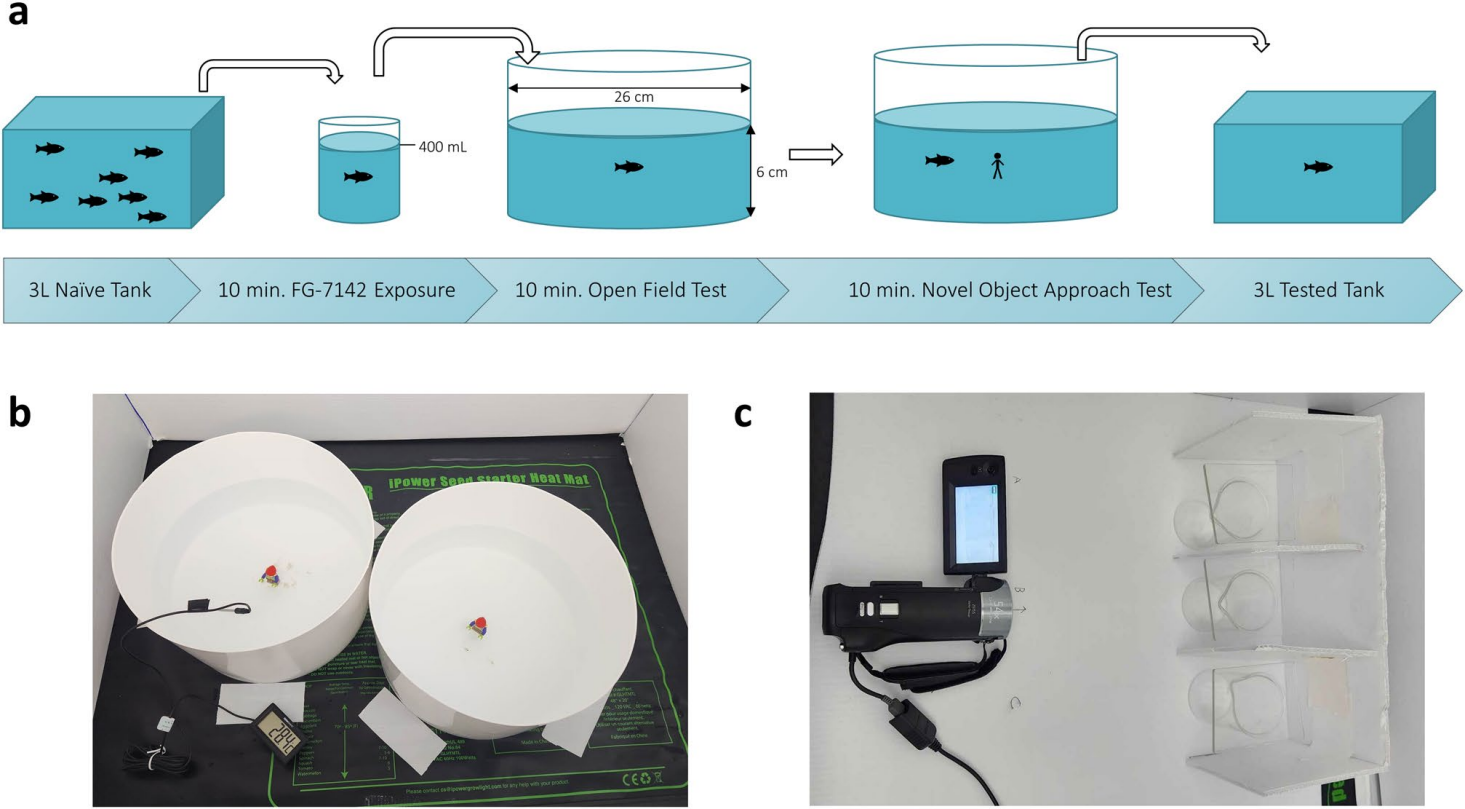
*Visualization of experimental setup and design.* The illustrated materials and procedure of the EC-response curve testing as well as arena placement. (**a**) Procedure and design of the OF-NOA testing. (**b**) Top-down demonstration of OF-NOA arena placement, stimuli, and temperature reading. (**c**) Top-down demonstration of opercular movement testing.

The OF procedure was based on previous work by Szaszkiewicz and colleagues^30^. After dosing, fish were netted individually into one of two separate white plastic circular testing arenas with a diameter of 26.0 cm, height of 11.0 cm, and a water depth of 6.0 cm (Fig. 8a). Both testing arenas were surrounded by four walls of corrugated plastic to reduce behavioural change due to visual stimuli outside of the testing apparatus. *n* = 16 fish from the same respective group were assessed on each testing day and *n* = 4 control fish were interspersed evenly throughout the trials. Final sample sizes were achieved after 4 testing days. Netted fish were released at the center of each arena and movement was recorded for 10 min. before the subsequent NOA (Fig. 8a). Locomotion (distance moved, immobility, mobility, & high mobility) and place preference (center, middle, & far/thigmotaxis zones) were quantified as measures of zebrafish anxiety and exploratory behaviours. Significant findings in locomotion were further explored post hoc by quantifying states of mobility and high mobility in addition to immobility to better understand locomotion while in movement. The virtual zones for the place preference variables were created in the EthoVision XT software (v. 11.0, Noldus, Wageningen, NL) to have approximately equal widths of 4.3 cm for the thigmotaxis zone (arena wall to middle zone edge), 4.3 cm for the middle zone (thigmotaxis zone edge to center zone edge), and 8.6 cm in diameter for the center zone (all area inside of the inner middle zone edge), as seen in Fig. 8a. Immobility was set to a threshold of 5% in which fish were considered to be immobile at any time fewer than 5% of the pixels of the fish’s body changed position. The high mobility threshold was similarly set for > 60% of pixels. The field of vision of the motion tracking camera allowed for the recording of two testing arenas simultaneously (Fig. 8b).

Following the OF, a 2 × 4.25 cm LEGO figurine was fixed to the center of each arena via a 1 × 2 LEGO brick adhered to the bottom of the arena to act as novel objects^54^. Each figurine was identical in shape, size, colour, and orientation. Fish movement was recorded for another 10 min. period and the same locomotor and place preference variables in the OF were quantified as measures of NOA anxiety and approach behaviour. Upon completion of the 10 min. period, fish were netted out of each arena and placed into a new 3L habitat tank, not to be tested again. The process was then repeated from the dosing step onward over 3 additional testing days until each *n* = 16 concentration group and *n* = 16 controls had been tested. Any residual FG-7142 present on the bodies of the exposed zebrafish was considered to be highly diluted in the testing arena water and of negligible risk to be discarded via the laboratory sink.

#### Open field test to open field test

To examine any anxiogenic or related effects found in repeated testing in the same arena with the the OF-NOA procedure, the OF-NOA procedure was replicated exactly, apart from the NOA being replaced with a second, identical OF. Another *n* = 16 fish were tested with the EC and another *n* = 16 control fish were tested for direct comparison. Following the completion of the first OF, another 10 min. trial was started without the introduction of the novel objects. The first OF, in this case, was to serve as an acclimation period for the second OF, and if similar NOA effects were seen during the second OF, then such effects could not be considered stimulus-dependent. If no effects were found in the second OF, then it could be determined that the EC effects are stimulus-dependent and that fish in this measure were acclimated to their environment.

#### Ethanol challenge testing

To compare the effects of the EC against a fear-reducing compound, the OF-NOA procedure was repeated with two additional groups of *n* = 16 each. The first additional group was treated only with 0.8% ethanol (3.2 mL_*EtOH*_ + 396.8 mL_*water*_), as one of the most commonly employed anxiolytics and fear-reducing compounds in behavioural research^50,54,56–58^. This EtOH group served as a baseline measure of the effects of ethanol on zebrafish alone. The second additional group was treated with both the EC of FG-7142 as well as the 0.8% EtOH concentration (EtOH + FG-7142). Both groups were combined with the OF-NOA controls and EC fish and compared for behavioural differences.

#### Opercular movement testing

To investigate any physiological changes to respiration rate with FG-7142, three groups of *n* = 10 fish (*n* = 30 total) were selectively sexed and randomly assigned as before. Three 100 mL beakers were prepared with either habitat water alone (100 mL_*water*_), habitat water containing the EC (75.25 µL_*FG-7142*_ + 99.9 mL_*water*_), or habitat water containing 0.8% ethanol (800 µL_*EtOH*_ + 99.2 mL_*water*_) to act as the sedative anxiolytic. Each beaker was covered with sheets of clear acrylic to prevent fish from escaping the water. A 9.2-megapixel Sony Handycam HDR-CX240 digital high-definition camcorder (Sony Electronics Inc., San Diego, CA, USA) was placed 25 cm away from the beakers, which were arranged centrally and perpendicular to the camera, equally distanced apart with white corrugated plastic dividing each beaker (Fig. 8c) to avoid visual confounds noted previously. Fish from each group were transferred into their respective beakers and recorded for 10 min. The beaker size, spacing, and distance from the camera were all arranged so that opercular movements would be visible upon later observation for manual quantification. Opercular beats-per-minute (BPM) were measured at 2-min intervals using a simple tap-tempo device, starting at 0:00 to yield opercular BPM for each fish at 0, 2, 4, 6, and 8 min. This collection strategy was useful, as data could be analyzed as mean trial BPM for each fish or mean BPM for all fish in each group at a given point in time. This was also considered more accurate than manually counting exact quantities of opercular movements for each fish given that the high frequency of movements and long durations of each trial made human error in manual counting extremely likely.

### Statistical analyses

All data were analyzed via either GraphPad Prism Software (Version 9.1.2; GraphPad, San Diego, CA, USA) or JASP (Version 0.17.1, JASP Team, 2023). A significance threshold of 5% (α = 0.05) and confidence interval of 95% were selected to determine statistical significance of all test results. All central tendency values were reported as means with variances reported as ± standard error in measurement (SEM). Any fish that were noted to have escaped the habitat tanks, dosing chambers, or testing arenas were excluded from analysis. Descriptive statistics (mean, median, SD, SEM, range, 95% CI) were calculated for each variable being measured. Normality was determined across group distributions on each variable via the D’Agostino-Pearson omnibus normality test and Bartlett’s test for equality of variances. Any test results across groups found to be significant indicated a normality violation and subsequent tests conducted were non-parametric or otherwise parametric. If significant differences were found in single-factor mean comparisons in either locomotion or place preference, a multifactor repeated measures ANOVA was used to investigate for main and interaction effects. All multifactor repeated measures ANOVA tests were conducted in the statistical analysis software JASP as GraphPad Prism is not appropriate for conducting repeated measures multifactor analyses unless at least two of only three variables have only two levels^59^. Any significant effect sizes would be interpreted as small (*η*^*2*^ ≤ 0.01), moderate (*η*^*2*^ = 0.06), or large (*η*^*2*^ ≥ 0.14).

For the OF-NOA analyses, two fish from the 10 µM group were excluded from analysis while the remainder were included in analysis for further testing. Exclusions yielded a population size of *n* = 78 zebrafish. Following subsequent normality tests, all locomotor (distance moved, immobility, mobility, & high mobility) and place preference (center, middle, & thigmotaxis) variables in each the OF and NOA were tested for significant differences using either a Brown-Forsythe one-way ANOVA for parametric data or an unpaired Kruskal–Wallis test for non-parametric data. If test results showed that a significant difference existed, parametric data was tested post hoc using Dunnett’s T3 multiple comparisons test and non-parametric data was tested post hoc using a Dunn’s multiple comparisons test. Group means of each variable were calculated along with their respective standard deviations, SEM, and 95% confidence intervals. Locomotor and place preference variables that showed significant differences would be investigated via a mixed model repeated measures 2 × 5x3 3-way ANOVA testing mean locomotion (test*concentration*distance moved/immobility/mobility/high mobility) or mean place preference (test*concentration*zone).

For the OF-OF analyses, no exclusions were deemed necessary, and all fish were included in analysis for further testing with a population size of *n* = 32 zebrafish for this validation measure. Following subsequent normality tests, all locomotor (distance moved, immobility, mobility, & high mobility) and place preference (center, middle, & thigmotaxis) variables in each OF were tested for significant differences using either a Welch’s t-Test for parametric data or a Mann–Whitney test for non-parametric data. Group means of each variable were calculated along with their respective standard deviations, SEM, and 95% confidence intervals. As the purpose of this testing was for validation of the OF-NOA findings, no further analyses were required for these data.

For the ethanol challenge analyses, the control group and the 10 µM group from the OF-NOA testing were compared to the EtOH and EtOH + FG-7142 groups. The same two fish from the OF-NOA 10 µM group were excluded from analyses while the remainder were included in analysis for further testing. Exclusions yielded a population size of *n* = 62 zebrafish for this measure. Following subsequent normality tests, all locomotor (distance moved, immobility, mobility, & high mobility) and place preference (center, middle, & thigmotaxis) variables in each the OF and NOA were tested for significant differences using either a Brown-Forsythe one-way ANOVA for parametric data or an unpaired Kruskal–Wallis test for non-parametric data. If test results showed that a significant difference existed, parametric data was tested post hoc using Dunnett’s T3 multiple comparisons test and non-parametric data was tested post hoc using a Dunn’s multiple comparisons test. Group means of each variable were calculated along with their respective standard deviations, SEM, and 95% confidence intervals. As the purpose of this testing was for validation of the OF-NOA findings, no further analyses were required for these data.

For the opercular movement analyses, no exclusions were deemed necessary, and all fish were included in analysis for further testing with a population size of *n* = 30 zebrafish for this measure. A Brown-Forsythe one-way ANOVA for parametric data or Kruskal–Wallis test for non-parametric data was used to determine whether significant differences existed between concentration groups for average opercular BPM. If test results showed that a significant difference existed, parametric data was tested post hoc using Dunnett’s T3 multiple comparisons test and non-parametric data was tested post hoc using a Dunn’s multiple comparisons test. Group means of each variable were calculated along with their respective standard deviations, SEM, and 95% confidence intervals. A repeated measures 3 × 5 two-way ANOVA was also used to investigate effects and effect sizes between concentration group (control vs. FG-7142 vs. EtOH) and time (0 vs. 2 vs. 4 vs. 6 vs. 8 min.) and post-hoc comparisons were explored using Tukey’s multiple comparisons to evaluate concentration groups for significant differences in average opercular BPM.

### Ethics declaration

All experiments carried out in this study were approved by MacEwan University’s Animal Research Ethics Board (AREB) under protocol number 101853 in compliance with the Canadian Council for Animal Care (CCAC) guidelines. All experiments were also in compliance with ARRIVE guidelines for animal research.

### Supplementary Information

Supplementary Information.

## Data Availability

All data collected throughout the experiments in this study are available via the supplemental materials document provided. Further inquiries can be directed to the corresponding author.
