# Supplementary material for: β-Carboline (FG-7142) modulates fear but not anxiety-like behaviour in zebrafish
Source: Sci Rep. 2024 Jan 5;14:668. doi: 10.1038/s41598-023-51072-6 (PMC10770314; doi:10.1038/s41598-023-51072-6)
Supplement: Supplementary file 1 — Supplementary Information. [file 41598_2023_51072_MOESM1_ESM.docx]

**β-Carboline (FG-7142) Modulates Fear but not Anxiety-like Behaviour in Zebrafish**

**SUPPLEMENTAL MATERIALS**

**Figures**


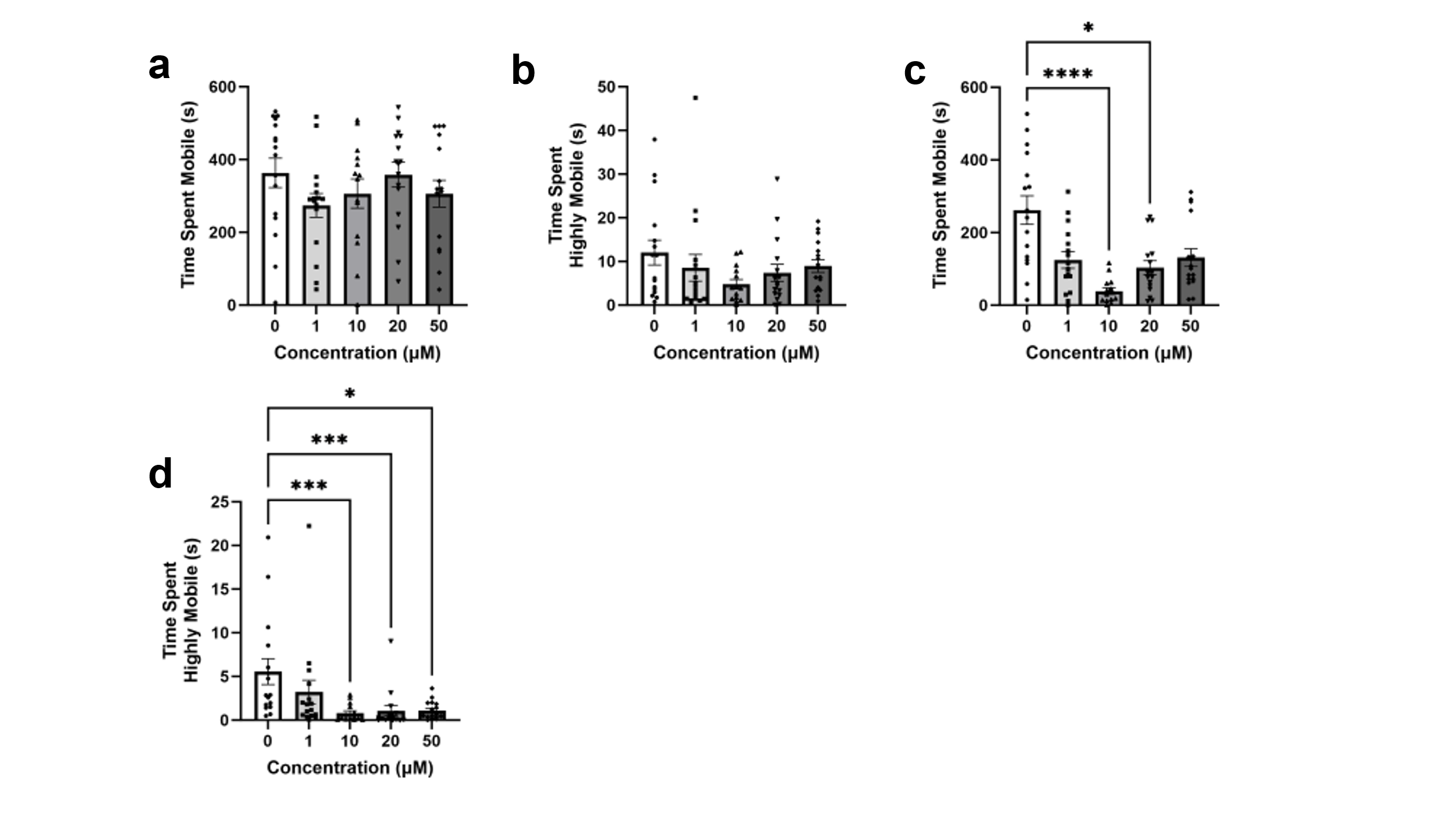


**Figure 1:** *Time spent mobile and highly mobile during the OF and NOA of the dose-response testing.* (A) Average time spent mobile by concentration group during the OF. (B) Average time spent highly mobile by concentration group during the OF. (C) Average time spent mobile by concentration group during the NOA. (D) Average time spent highly mobile by concentration group during the NOA. Error bars represent S.E.M. **p* < 0.05, ****p* <0.001, *****p* < 0.0001.


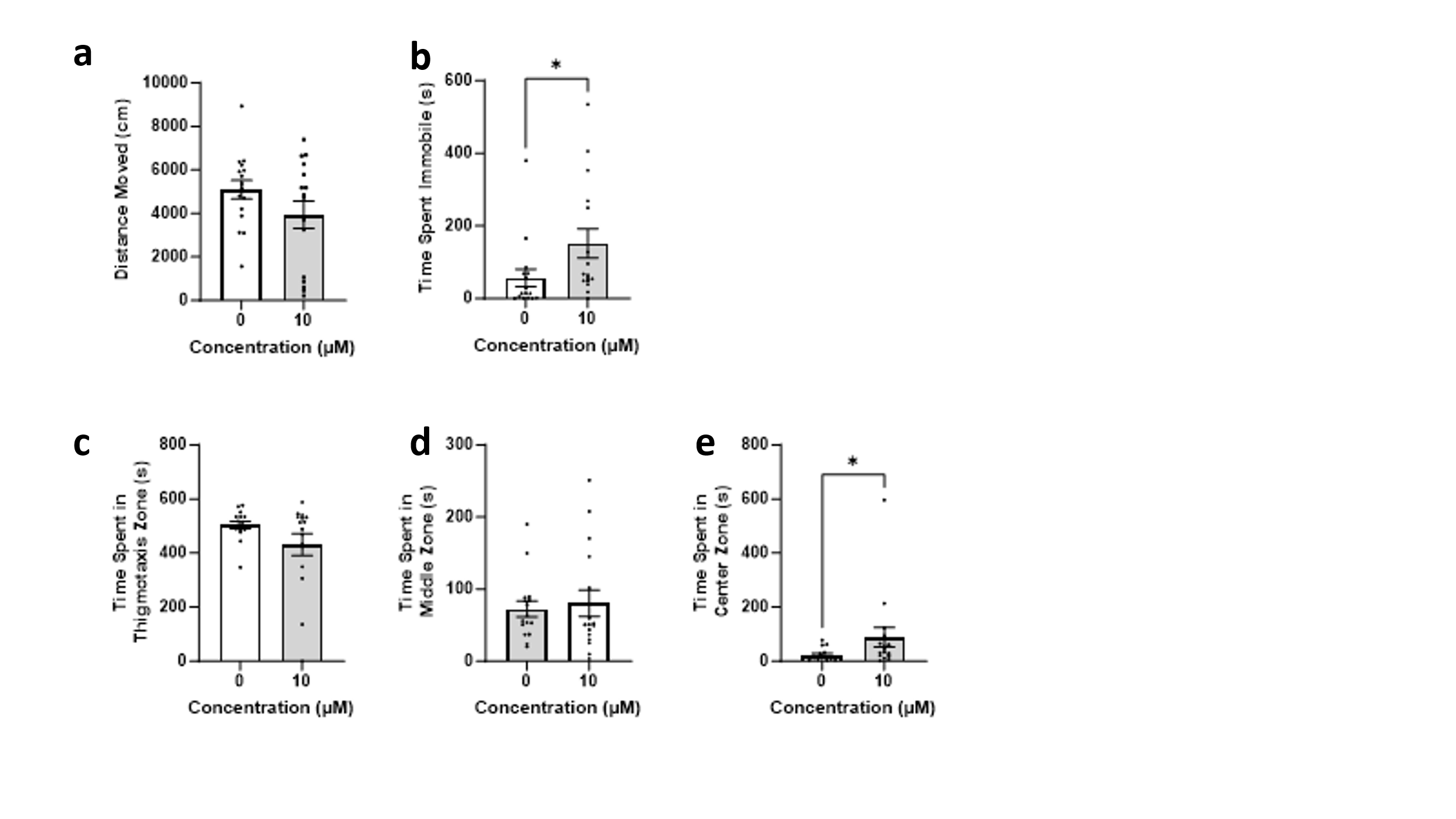


**Figure 2:** *First open field test locomotion and place preference during the open field test to open field test validation.* Zebrafish locomotor and place preference responses during the OF-OF validation testing. (A) Average distance moved by concentration group. (B) Average time spent immobile by concentration group. (C) Average time spent in the thigmotaxis zone by concentration group. (D) Average time spent in the middle zone by concentration group. (E) Average time spent in the center zone by concentration group. Error bars represent S.E.M. **p* < 0.05.


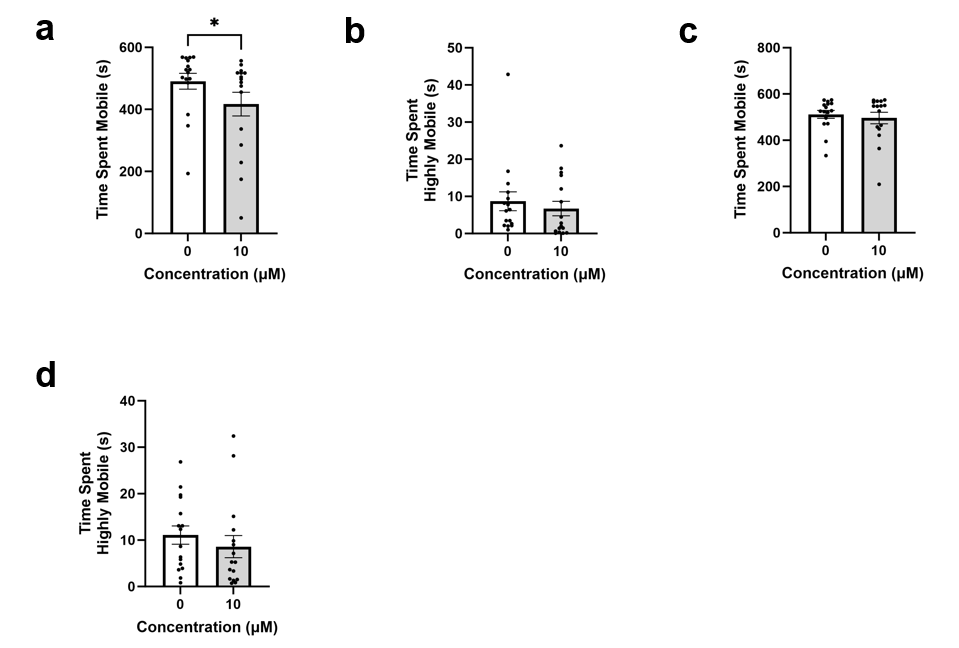


**Figure 3:** *Time spent mobile and highly mobile during each OF of the repeated OF testing.* (A) Average time spent mobile by concentration group during the first OF. (B) Average time spent highly mobile by concentration group during the first OF. (C) Average time spent mobile by concentration group during the second OF. (D) Average time spent highly mobile by concentration group during the second OF. Error bars represent S.E.M. **p* < 0.05.


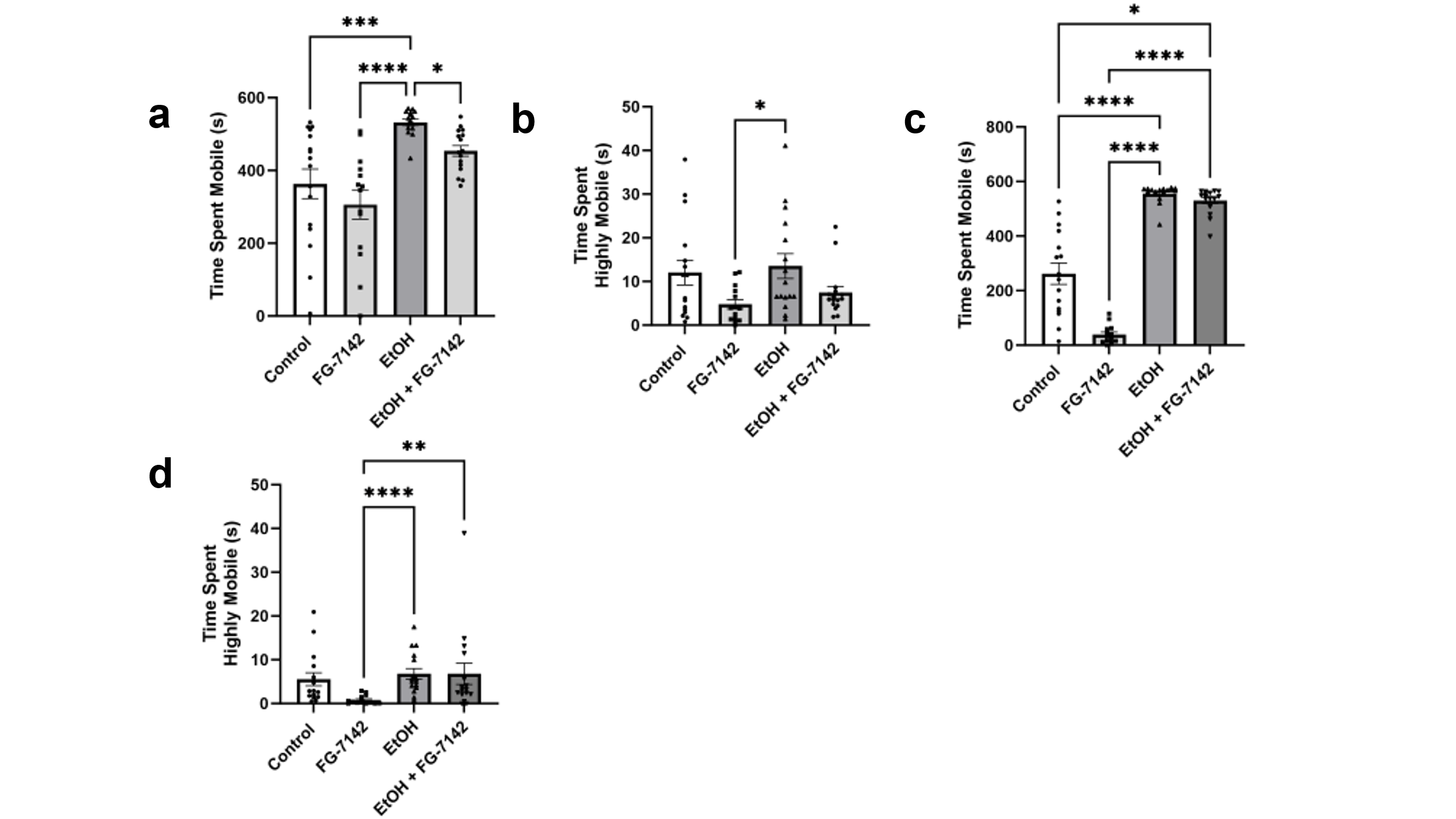


**Figure 4:** *Time spent mobile and highly mobile during the ethanol challenge testing.* (A) Average time spent mobile by group during the OF. (B) Average time spent highly mobile by group during first OF. (C) Average time spent mobile by group during the NOA. (D) Average time spent highly mobile by group during the NOA. Error bars represent S.E.M. **p* < 0.05, ***p* < 0.01, ****p* < 0.001, *****p* < 0.0001.

**Figure 5:** *Post-hoc comparison of response curve testing groups for group-specific center zone preference during the OF vs. NOA.* Average time spent in the center zone by groups when in either the OF or NOA. Error bars represent S.E.M.

*Note:* The following data points fall outside of the y-axis as outliers, but did not significantly impact the significance of the results – NOA [10 µM = 599.72 s and 304.76 s] and OF [0 µM = 238.48 s]. These were omitted in this figure for visual comprehension of the results.

**Within Subjects Effects**

| **Cases** | **Sum of Squares** | **df** | **Mean Square** | **F** | ***p*** |
| --- | --- | --- | --- | --- | --- |
| Concentration*Test | 4862 | 4 | 1215 | 0.9032 | 0.4639 |
| Concentration | 4441 | 4 | 1110 | 0.8250 | 0.5112 |
| Test | 528.4 | 1 | 528.4 | 0.3926 | 0.5319 |
| Residuals | 195138 | 145 | 1346 |  |  |

*Note.* Type III Sum of Squares

**Table 1:** *Open Field Test to Novel Object Approach Test Post-Hoc Comparison of Center Zone Preference Within Concentration Groups Between Test Periods.*

**Results**

**Open Field Test**

***Open Field Test - Locomotion***

Post hoc results of mobility (Supp. Fig. 1a, *F*(4, 70.84) = 1.093, *p* = 0.3666) and high mobility (Supp. Fig. 1b, *H*(4) = 5.953, *p* = 0.2027) were also not significantly different.

**Novel Object Approach Test**

***Novel Object Approach Test - Locomotion***

Post hoc, a significant difference was found between concentration groups during the NOA for time spent mobile (Supp. Fig. 1c, *F*(4, 46.31) = 10.28, *p* < 0.0001) and Dunnett’s T3 multiple comparisons found significant differences between the 1 µM (*µ* = 124.4s, *SEM* = 22.69, *p* = 0.0217), 10 µM (*µ* = 38.84 s, *SEM* = 9.190, *p* = 0.0001), 20 µM (*µ* = 103.7 s, *SEM* = 19.24, *p* = 0.0058), and 50 µM (*µ* = 131.2 s, *SEM* = 24.73, *p* = 0.0351) fish vs. the 0 µM control group (*µ* = 262.0 s, *SEM* = 39.05). A significant difference was also found between concentration groups during the NOA for time spent highly mobile (Supp. Fig. 1d, *H*(4) = 24.04, *p* < 0.0001) and Dunnett’s T3 multiple comparisons found significant differences between the 10 µM (*µ* = 27.89 s, *SEM* = 0.2647, *p* = 0.0005), 20 µM (*µ* = 26.03 s, *SEM* = 0.5708, *p* = 0.0001), and 50 µM (*µ* = 35.91 s, *SEM* = 0.2610, *p* = 0.0116) fish vs. the 0 µM control group (*µ* = 59.75 s, *SEM* = 1.476).

**Post-Hoc Multifactor Analysis**

Following the post-hoc multifactor analysis, a further comparison was made between individual groups for center zone preference during the OF vs. the NOA to evaluate each group for changes in preference for or away from the center zone between each test. A repeated measures 2-way ANOVA was used as residual analysis confirmed that assumptions of normality, homogeneity, and homoscedasticity were satisfied, and the model was appropriate.

No significant main effects were found for concentration (*F*(4, 145) = 0.8250, *p* = 0.4639) or test (*F*(4, 145) = 0.3926, *p* = 0.5319) nor was there a significant interaction between the two variables for within-group center zone preference (concentration*test, *F*(4, 145) = 0.9032, *p* = 0.4639).

In summary, individually, concentration groups did not appear to show any significant change in preference for or against the center zone when comparing the OF against the NOA time spent near the novel object. These results can be seen in Supplemental Figure 5 and Supplemental Table 1.

**Repeated Open Field Test**

***First Open Field Test***

A significant difference was found between concentration groups for time spent mobile (Supp. Fig. 3a, *U* = 75, *p* = 0.0468) in which 10 µM fish (*Mdn* = 50.12 s) spent significantly less time mobile than 0 µM fish (*Mdn* = 193.0 s). No significant difference was found between concentration groups for time spent highly mobile (Supp. Fig. 3b, *U* = 127, *p* = 0.9852).

***Second Open Field Test***

No significant differences were found between concentration groups for time spent mobile (Supp. Fig. 3c, *U* = 127, *p* = 0.9852) or time spent highly mobile (Supp. Fig. 3d, *U* = 93, *p* = 0.1930).

**Ethanol Challenge**

***Ethanol – Open Field Test***

A significant difference was found between groups for time spent mobile (Supp. Fig. 4a, *H*(3) = 30.91, *p* < 0.0001) and Dunn’s multiple comparisons showed significant differences between the EtOH group (*µ* = 532.3 s, *SEM* = 8.707) and the control group (*µ* = 363.4 s, *SEM* = 40.48, *p* = 0.0003), EtOH+FG-7142 group (*µ* = 453.8 s, *SEM* = 14.59, *p* = 0.0174), and FG-7142 group (*µ* = 306.4 s, *SEM* = 40.14, *p* < 0.0001). No significant differences were found between groups for time spent highly mobile (Supp. Fig. 4b, *H*(3) = 7.788, *p* = 0.0506), but because of proximity to significance, Dunn’s multiple comparisons were used and found a significant difference between the FG-7142 group (*µ* = 4.774 s, *SEM* = 1.087) and the EtOH group (*µ* = 13.53 s, *SEM* = 2.827, *p* = 0.0390).

***Ethanol – Novel Object Approach Test***

A significant difference was found between groups for time spent mobile (Supp. Fig. 4c, *H*(3) = 50.09, *p* < 0.0001) and Dunn’s multiple comparisons showed significant differences between the EtOH group (*µ* = 555.3 s, *SEM* = 8.448) and both the control group (*µ* = 262.0 s, *SEM* = 39.05, *p* < 0.0001) and FG-7142 group (*µ* = 38.84 s, *SEM* = 9.190, *p* < 0.0001), as well as between the EtOH+FG-7142 group (*µ* = 530.4 s, *SEM* = 11.51) and both the FG-7142 group (*µ* = 38.84 s, *SEM* = 9.190, *p* < 0.0001) and control group (*µ* = 262.0 s, *SEM* = 39.05, *p* = 0197). A significant difference was found between groups for time spent highly mobile (Supp. Fig. 4d, *H*(3) = 21.46, *p* < 0.0001) and Dunn’s multiple comparisons found significant differences between the FG-7142 group (*µ* = 0.8143 s, *SEM* = 0.2647) and the EtOH+FG-7142 group (*µ* = 6.783 s, *SEM* = 2.431, *p* = 0.0089), EtOH group (*µ* = 6.795 s, *SEM* = 2.431, *p* < 0.0001), and control group (*µ* = 5.550 s, *SEM* = 1.746, *p* = 0.0058).

**Data**

***OF-NOA – Locomotion***

| OF - Distance Moved (cm) | | | | |
| --- | --- | --- | --- | --- |
| 0 | 1 | 10 | 20 | 50 |
| 7987.30 | 9198.20 | 4337.80 | 9828.04 | 1255.68 |
| 6534.13 | 9155.68 | 5905.52 | 5707.82 | 6712.26 |
| 5495.34 | 5976.48 | 5275.74 | 6461.65 | 9314.47 |
| 8239.49 | 4850.06 | 5778.83 | 6807.71 | 9723.52 |
| 4369.52 | 5670.44 | 5305.64 | 6900.00 | 7117.83 |
| 8678.79 | 1399.07 | 6304.60 | 7871.09 | 5719.02 |
| 4513.12 | 3165.51 | 131.85 | 7251.69 | 6274.26 |
| 9846.41 | 5856.01 | 6026.64 | 8734.65 | 9539.37 |
| 6563.54 | 6308.15 | 8713.96 | 6597.07 | 3958.61 |
| 8024.52 | 5502.29 | 7669.88 | 6062.45 | 9411.54 |
| 8727.71 | 1045.56 | 1953.75 | 7874.67 | 3839.94 |
| 6262.38 | 5897.78 | 9106.96 | 1391.50 | 2571.57 |
| 8144.39 | 4998.92 | 6690.43 | 4834.46 | 6206.16 |
| 9643.04 | 4833.14 | 8434.40 | 4566.99 | 7252.35 |
| 409.23 | 5857.06 |  | 2381.85 | 5924.38 |
| 4056.79 | 6245.44 |  | 7356.23 | 5999.73 |

| NOA - Distance Moved (cm) | | | | |
| --- | --- | --- | --- | --- |
| 0 | 1 | 10 | 20 | 50 |
| 5705.48 | 741.71 | 2702.76 | 5218.28 | 4183.76 |
| 5671.94 | 4877.74 | 1538.85 | 3541.38 | 3156.47 |
| 4204.36 | 1777.49 | 655.99 | 3411.85 | 6627.84 |
| 5598.11 | 4418.67 | 2877.11 | 2546.14 | 737.82 |
| 3494.82 | 4159.63 | 3398.29 | 3227.41 | 539.93 |
| 8828.60 | 1824.87 | 1402.54 | 3731.59 | 3352.97 |
| 4657.21 | 3060.06 | 222.49 | 2844.76 | 6229.73 |
| 7859.24 | 4751.73 | 1528.06 | 4363.37 | 6971.50 |
| 4253.52 | 4732.61 | 793.85 | 3064.13 | 3373.89 |
| 6734.38 | 4235.05 | 895.02 | 1329.04 | 3251.00 |
| 2380.10 | 387.33 | 350.02 | 5702.89 | 4465.08 |
| 4518.49 | 2991.99 | 719.13 | 453.81 | 3029.25 |
| 5681.66 | 3981.81 | 4369.75 | 2977.48 | 2604.48 |
| 8404.73 | 27.41 | 611.00 | 3206.43 | 3143.85 |
| 534.75 | 2928.24 |  | 913.21 | 4306.40 |
| 1169.10 | 944.22 |  | 1284.77 | 6180.51 |

| OF - Immobility (s) | | | | |
| --- | --- | --- | --- | --- |
| 0 | 1 | 10 | 20 | 50 |
| 93.60 | 53.04 | 395.56 | 46.92 | 547.24 |
| 208.04 | 52.24 | 228.12 | 269.76 | 244.88 |
| 289.80 | 268.60 | 230.52 | 194.12 | 89.08 |
| 117.32 | 306.76 | 243.44 | 253.12 | 40.48 |
| 430.44 | 219.08 | 350.20 | 112.48 | 239.40 |
| 112.88 | 526.00 | 231.44 | 152.44 | 329.64 |
| 400.12 | 468.56 | 592.56 | 121.28 | 266.32 |
| 26.72 | 260.60 | 302.92 | 31.20 | 82.84 |
| 261.16 | 223.80 | 80.40 | 230.96 | 433.08 |
| 93.24 | 281.36 | 192.72 | 198.52 | 76.08 |
| 65.32 | 549.04 | 520.00 | 118.16 | 441.16 |
| 149.60 | 293.40 | 89.72 | 524.76 | 487.24 |
| 67.52 | 389.52 | 186.92 | 340.00 | 277.64 |
| 59.68 | 300.16 | 163.64 | 367.80 | 141.16 |
| 582.72 | 266.32 |  | 470.48 | 250.88 |
| 339.96 | 237.96 |  | 183.40 | 285.52 |

| NOA - Immobility (s) | | | | |
| --- | --- | --- | --- | --- |
| 0 | 1 | 10 | 20 | 50 |
| 111.20 | 556.60 | 507.24 | 358.44 | 505.60 |
| 221.52 | 315.48 | 543.40 | 528.16 | 499.76 |
| 359.88 | 505.08 | 579.60 | 472.56 | 332.96 |
| 264.40 | 279.08 | 495.88 | 496.88 | 577.52 |
| 414.36 | 383.20 | 507.36 | 455.44 | 573.24 |
| 48.44 | 504.88 | 553.88 | 449.32 | 511.28 |
| 393.40 | 499.96 | 593.24 | 457.72 | 262.88 |
| 136.20 | 359.44 | 578.32 | 351.08 | 228.24 |
| 475.72 | 356.00 | 565.32 | 537.44 | 387.44 |
| 172.64 | 396.44 | 568.08 | 546.48 | 484.72 |
| 463.44 | 581.36 | 593.36 | 347.48 | 456.52 |
| 348.56 | 451.16 | 586.64 | 576.92 | 450.28 |
| 268.32 | 453.60 | 484.16 | 516.16 | 526.76 |
| 100.44 | 584.52 | 436.76 | 503.12 | 482.44 |
| 576.00 | 477.12 |  | 567.72 | 435.24 |
| 531.40 | 563.20 |  | 558.32 | 266.04 |

| OF - Mobility (s) | | | | |
| --- | --- | --- | --- | --- |
| 0 | 1 | 10 | 20 | 50 |
| 412.48 | 493 | 170.4 | 514.76 | 42.96 |
| 356.28 | 517.92 | 360.64 | 320.16 | 319.44 |
| 250.68 | 290.52 | 280.88 | 395.2 | 491.28 |
| 458.16 | 275.12 | 345.84 | 330.4 | 492.32 |
| 105.52 | 293.48 | 188.88 | 474.88 | 340.6 |
| 451.88 | 60.76 | 356.36 | 431.72 | 188.08 |
| 192.76 | 105.2 | 0 | 465.24 | 320.24 |
| 532.2 | 330.44 | 287.12 | 544 | 468.44 |
| 327.92 | 294.2 | 507.8 | 360.6 | 153.08 |
| 494.32 | 310.24 | 403.44 | 395.12 | 491.92 |
| 512.12 | 43.44 | 79.16 | 464.44 | 146.6 |
| 433.6 | 291.52 | 499.44 | 65.6 | 89.08 |
| 519.88 | 172.44 | 385.72 | 250.04 | 308 |
| 520.16 | 286.92 | 424.08 | 214.96 | 429.88 |
| 6.72 | 263.72 |  | 118.04 | 312.4 |
| 239.04 | 352.4 |  | 388.6 | 301.36 |

| NOA - Mobility (s) | | | | |
| --- | --- | --- | --- | --- |
| 0 | 1 | 10 | 20 | 50 |
| 261.04 | 34.16 | 62.2 | 234 | 83.24 |
| 357.6 | 254.84 | 44.2 | 64.32 | 61.64 |
| 163.16 | 79.28 | 11.84 | 119.32 | 260.76 |
| 325.68 | 312.52 | 96.6 | 92.04 | 16.4 |
| 133.68 | 169.2 | 59.04 | 137 | 17.64 |
| 526.84 | 79.92 | 39.64 | 142.16 | 72.16 |
| 201.84 | 86.36 | 0.24 | 78.52 | 311.92 |
| 442.56 | 233.24 | 15.48 | 234.08 | 285.32 |
| 115.88 | 150.12 | 34.32 | 54.64 | 83.8 |
| 419.08 | 195.36 | 29.36 | 45.32 | 101.04 |
| 124.64 | 11.88 | 4.32 | 244 | 134.52 |
| 241 | 138.48 | 13.36 | 13.56 | 72.8 |
| 322.44 | 116.24 | 115.56 | 77.84 | 66.12 |
| 482.88 | 0.6 | 17.64 | 90.16 | 108.44 |
| 14.56 | 99.32 |  | 11.64 | 133.16 |
| 58.92 | 29.04 |  | 21.2 | 291 |

| OF – High Mobility (s) | | | | |
| --- | --- | --- | --- | --- |
| 0 | 1 | 10 | 20 | 50 |
| 18.28 | 47.48 | 1.08 | 28.92 | 2.16 |
| 29.76 | 21.56 | 4.28 | 0.16 | 16.56 |
| 3.6 | 19.44 | 3.92 | 2.68 | 11.36 |
| 14.76 | 10.36 | 2.52 | 7.96 | 19.2 |
| 1.72 | 1.48 | 1.68 | 4.52 | 10.56 |
| 28.36 | 4.96 | 6.56 | 6.44 | 0.92 |
| 6.2 | 1 | 0 | 5 | 6.36 |
| 37.92 | 1.48 | 1.36 | 15.12 | 17.44 |
| 0.76 | 6.36 | 11.8 | 2.36 | 5.88 |
| 2.16 | 2.44 | 3.8 | 0.28 | 9.28 |
| 13.4 | 1.44 | 0.8 | 6.16 | 3.44 |
| 5.64 | 9.16 | 9.12 | 1.4 | 14.4 |
| 4.16 | 0.56 | 7.8 | 3.44 | 6.44 |
| 11.48 | 4.4 | 12.12 | 10.68 | 12.4 |
| 2.92 | 3.4 |  | 3.68 | 4.04 |
| 11.44 | 1.08 |  | 19.72 | 3.32 |

| NOA – High Mobility (s) | | | | |
| --- | --- | --- | --- | --- |
| 0 | 1 | 10 | 20 | 50 |
| 6.04 | 1.8 | 0 | 0.04 | 0.12 |
| 10.64 | 22.24 | 1.48 | 0 | 0.44 |
| 2.84 | 5.72 | 0.2 | 0.2 | 0.52 |
| 2.6 | 2 | 0.8 | 3.16 | 0.36 |
| 1.96 | 2.4 | 0.04 | 0.68 | 0.48 |
| 16.4 | 6.52 | 0.6 | 1.64 | 0.64 |
| 4.76 | 1.16 | 0.4 | 0.6 | 0 |
| 20.92 | 0.6 | 0.08 | 9.08 | 2 |
| 0.68 | 0.52 | 0.2 | 0 | 1.24 |
| 0.48 | 1.88 | 2.56 | 0.28 | 1.84 |
| 5.36 | 0.4 | 1.92 | 0.08 | 1.96 |
| 2.88 | 4.16 | 0 | 0.84 | 3.64 |
| 1.4 | 0.64 | 0.24 | 0.24 | 0.36 |
| 8.56 | 0.08 | 2.88 | 0.08 | 1.4 |
| 1.52 | 1 |  | 0.28 | 0.2 |
| 1.76 | 0.36 |  | 0.12 | 2.6 |

***OF-NOA – Place Preference***

| OF - Time in Center Zone (s) | | | | |
| --- | --- | --- | --- | --- |
| 0 | 1 | 10 | 20 | 50 |
| 4.20 | 1.00 | 6.64 | 1.40 | 2.96 |
| 8.44 | 0.92 | 31.00 | 4.24 | 14.80 |
| 13.96 | 1.44 | 7.80 | 2.44 | 2.64 |
| 1.28 | 6.36 | 8.00 | 30.44 | 0.80 |
| 5.68 | 3.76 | 6.72 | 1.92 | 6.16 |
| 0.76 | 3.04 | 4.80 | 6.52 | 8.40 |
| 12.00 | 50.36 | 0.00 | 1.68 | 11.68 |
| 6.52 | 24.96 | 5.76 | 0.16 | 2.92 |
| 16.20 | 5.84 | 5.60 | 10.36 | 19.84 |
| 7.24 | 2.20 | 7.88 | 10.96 | 2.04 |
| 0.96 | 0.84 | 6.28 | 0.68 | 41.00 |
| 0.16 | 5.96 | 2.12 | 0.24 | 3.48 |
| 0.88 | 16.28 | 2.76 | 33.20 | 2.84 |
| 1.04 | 14.28 | 2.12 | 75.56 | 2.60 |
| 238.48 | 13.00 |  | 148.20 | 5.80 |
| 6.16 | 4.36 |  | 10.72 | 16.48 |

| NOA - Time in Center Zone (s) | | | | |
| --- | --- | --- | --- | --- |
| 0 | 1 | 10 | 20 | 50 |
| 1.24 | 0.00 | 0.00 | 0.00 | 5.76 |
| 6.92 | 0.00 | 0.00 | 0.00 | 0.00 |
| 19.64 | 0.00 | 1.16 | 0.00 | 0.16 |
| 1.64 | 0.28 | 0.00 | 0.28 | 0.00 |
| 3.76 | 10.76 | 0.60 | 0.36 | 0.00 |
| 0.00 | 0.20 | 1.68 | 5.32 | 0.00 |
| 1.24 | 1.32 | 599.72 | 0.00 | 0.00 |
| 0.84 | 4.28 | 0.12 | 0.00 | 0.16 |
| 0.68 | 4.00 | 304.76 | 2.68 | 0.04 |
| 7.04 | 0.00 | 1.36 | 169.80 | 1.40 |
| 0.00 | 0.16 | 2.20 | 2.36 | 2.76 |
| 1.64 | 0.16 | 2.16 | 1.88 | 2.52 |
| 2.76 | 0.28 | 11.96 | 0.00 | 0.40 |
| 1.88 | 13.68 | 0.00 | 1.00 | 1.84 |
| 0.00 | 1.44 |  | 78.28 | 6.80 |
| 0.00 | 0.00 |  | 0.96 | 5.16 |

| OF - Time in Middle Zone (s) | | | | |
| --- | --- | --- | --- | --- |
| 0 | 1 | 10 | 20 | 50 |
| 14.92 | 7.80 | 58.00 | 16.52 | 92.08 |
| 31.60 | 9.64 | 84.28 | 29.00 | 107.72 |
| 51.72 | 13.36 | 28.80 | 23.00 | 20.92 |
| 12.48 | 43.88 | 37.20 | 12.12 | 12.64 |
| 27.28 | 30.44 | 27.24 | 12.00 | 23.68 |
| 27.60 | 41.24 | 20.52 | 15.56 | 45.36 |
| 122.76 | 159.80 | 599.84 | 20.44 | 111.96 |
| 16.28 | 53.68 | 21.20 | 0.88 | 16.64 |
| 35.32 | 30.96 | 24.68 | 40.76 | 84.52 |
| 19.80 | 23.48 | 30.08 | 28.08 | 15.00 |
| 15.44 | 12.96 | 25.20 | 11.24 | 102.48 |
| 4.68 | 55.20 | 18.12 | 3.24 | 101.60 |
| 10.16 | 41.32 | 21.72 | 139.80 | 55.04 |
| 9.92 | 96.36 | 21.20 | 205.36 | 20.64 |
| 319.24 | 24.08 |  | 172.68 | 27.96 |
| 134.92 | 22.20 |  | 30.20 | 56.32 |

| NOA - Time in Middle Zone (s) | | | | |
| --- | --- | --- | --- | --- |
| 0 | 1 | 10 | 20 | 50 |
| 20.72 | 77.84 | 26.00 | 13.88 | 33.52 |
| 20.64 | 7.16 | 215.16 | 30.88 | 49.52 |
| 65.76 | 6.84 | 92.00 | 90.40 | 33.52 |
| 8.48 | 9.40 | 5.64 | 72.56 | 2.56 |
| 40.04 | 109.60 | 48.00 | 39.80 | 165.20 |
| 0.28 | 110.12 | 90.64 | 113.64 | 21.80 |
| 38.84 | 192.44 | 0.32 | 30.92 | 8.80 |
| 41.08 | 131.32 | 14.28 | 10.84 | 22.52 |
| 117.76 | 44.64 | 44.04 | 127.24 | 91.72 |
| 27.08 | 45.64 | 347.20 | 137.80 | 51.92 |
| 4.04 | 2.04 | 594.16 | 95.84 | 41.76 |
| 40.60 | 7.96 | 197.00 | 3.40 | 59.92 |
| 61.68 | 59.48 | 83.24 | 102.24 | 82.68 |
| 16.84 | 0.16 | 6.92 | 60.12 | 49.28 |
| 485.24 | 19.92 |  | 301.40 | 81.52 |
| 83.76 | 184.64 |  | 67.04 | 30.20 |

| OF - Time in Thigmotaxis Zone (s) | | | | |
| --- | --- | --- | --- | --- |
| 0 | 1 | 10 | 20 | 50 |
| 580.36 | 591.24 | 535.40 | 582.12 | 505.00 |
| 560.00 | 589.48 | 484.76 | 566.80 | 477.52 |
| 534.36 | 585.24 | 563.44 | 574.60 | 576.48 |
| 586.28 | 549.80 | 554.84 | 557.48 | 586.60 |
| 567.08 | 565.84 | 566.08 | 586.12 | 570.16 |
| 571.68 | 555.76 | 574.72 | 577.96 | 546.24 |
| 465.28 | 389.84 | 0.20 | 577.92 | 476.40 |
| 577.24 | 521.36 | 573.08 | 599.00 | 580.48 |
| 548.52 | 563.24 | 569.76 | 548.92 | 495.68 |
| 573.00 | 574.36 | 562.08 | 561.00 | 583.00 |
| 583.64 | 580.16 | 568.56 | 588.00 | 456.56 |
| 595.20 | 537.52 | 579.80 | 596.56 | 494.96 |
| 589.00 | 542.44 | 565.12 | 427.04 | 542.16 |
| 589.08 | 489.40 | 576.72 | 319.12 | 576.80 |
| 42.32 | 562.96 |  | 279.16 | 566.28 |
| 458.96 | 573.48 |  | 559.12 | 527.24 |

| NOA - Time in Thigmotaxis Zone (s) | | | | |
| --- | --- | --- | --- | --- |
| 0 | 1 | 10 | 20 | 50 |
| 578.08 | 522.20 | 574.04 | 586.16 | 560.76 |
| 572.48 | 592.88 | 384.88 | 569.16 | 550.52 |
| 514.64 | 593.20 | 506.88 | 509.64 | 566.36 |
| 589.92 | 590.36 | 594.40 | 527.20 | 597.48 |
| 556.24 | 479.64 | 551.44 | 559.88 | 434.84 |
| 599.76 | 489.72 | 507.72 | 481.08 | 578.24 |
| 559.96 | 406.24 | 0.00 | 569.12 | 591.24 |
| 558.12 | 464.44 | 585.64 | 589.20 | 577.36 |
| 481.60 | 551.40 | 251.24 | 470.12 | 508.24 |
| 565.92 | 554.40 | 251.48 | 292.44 | 546.72 |
| 596.00 | 597.84 | 3.68 | 501.84 | 555.52 |
| 557.80 | 591.92 | 400.88 | 594.76 | 537.60 |
| 535.60 | 540.28 | 504.84 | 497.80 | 516.96 |
| 581.32 | 2.68 | 593.12 | 538.92 | 548.92 |
| 114.80 | 578.68 |  | 220.36 | 511.72 |
| 516.28 | 415.40 |  | 532.04 | 564.68 |

***Repeated OF – Locomotion***

| OF 1 - Distance Moved (cm) | |
| --- | --- |
| 0 | 10 |
| 6231.38 | 5789.14 |
| 5719.51 | 1051.45 |
| 5976.83 | 7395.06 |
| 4792.15 | 5182.01 |
| 4716.27 | 6689.90 |
| 8938.19 | 4724.43 |
| 3887.12 | 210.87 |
| 5361.56 | 462.98 |
| 4205.21 | 602.26 |
| 6418.48 | 5180.99 |
| 6381.68 | 3265.49 |
| 5929.09 | 6263.43 |
| 5161.92 | 6649.89 |
| 3124.80 | 866.25 |
| 1561.41 | 4842.18 |
| 3103.50 | 3723.85 |

| OF 2 - Distance Moved (cm) | |
| --- | --- |
| 0 | 10 |
| 5641.57 | 3908.13 |
| 5034.49 | 1359.50 |
| 4870.85 | 8133.82 |
| 4931.34 | 5557.64 |
| 3846.26 | 8469.44 |
| 7127.31 | 4778.65 |
| 4568.85 | 1107.98 |
| 3880.35 | 1785.40 |
| 5530.55 | 738.60 |
| 7251.32 | 7243.34 |
| 6402.85 | 1974.64 |
| 5686.67 | 6173.38 |
| 4889.92 | 5504.31 |
| 4617.03 | 957.24 |
| 1983.68 | 3950.52 |
| 3275.31 | 6074.12 |

| OF 1 - Immobility (s) | |
| --- | --- |
| 0 | 10 |
| 0.16 | 0.48 |
| 0.24 | 268.92 |
| 4.20 | 55.00 |
| 68.08 | 96.44 |
| 0.80 | 67.28 |
| 14.20 | 63.52 |
| 69.00 | 534.64 |
| 1.64 | 352.76 |
| 85.40 | 405.64 |
| 13.80 | 48.52 |
| 56.32 | 127.56 |
| 13.56 | 53.76 |
| 2.48 | 17.88 |
| 165.44 | 249.04 |
| 379.68 | 38.80 |
| 28.24 | 47.80 |

| OF 2 - Immobility (s) | |
| --- | --- |
| 0 | 10 |
| 0.32 | 14.96 |
| 0.80 | 359.00 |
| 1.24 | 0.12 |
| 22.04 | 0.68 |
| 1.12 | 1.32 |
| 4.08 | 2.12 |
| 7.12 | 108.04 |
| 48.52 | 154.08 |
| 35.56 | 124.92 |
| 1.36 | 0.84 |
| 0.36 | 116.00 |
| 0.40 | 2.40 |
| 1.08 | 0.92 |
| 3.00 | 221.24 |
| 100.88 | 1.32 |
| 29.92 | 0.88 |

| OF 1 - Mobility (s) | |
| --- | --- |
| 0 | 10 |
| 502.6 | 556.88 |
| 568.24 | 285.2 |
| 528.12 | 487.12 |
| 497.84 | 475 |
| 383.44 | 497.56 |
| 527.6 | 517.2 |
| 498.92 | 50.12 |
| 568.92 | 228.92 |
| 485.8 | 174.56 |
| 557.16 | 517.52 |
| 518.96 | 455.56 |
| 565.72 | 517.12 |
| 567.48 | 543.76 |
| 347.28 | 336.64 |
| 193.04 | 524.16 |
| 538.56 | 504.24 |

| OF 2 - Mobility (s) | |
| --- | --- |
| 0 | 10 |
| 395.04 | 548.4 |
| 542.48 | 209.64 |
| 496.12 | 526.28 |
| 527.96 | 568.88 |
| 333.56 | 547.56 |
| 558.88 | 572.76 |
| 552.32 | 464.84 |
| 525.52 | 421.48 |
| 523.72 | 449 |
| 557.04 | 546.8 |
| 574.56 | 457.44 |
| 567.92 | 569.28 |
| 573.64 | 550.32 |
| 471.4 | 364.36 |
| 471.08 | 574.32 |
| 520.12 | 564.96 |

| OF 1 – High Mobility (s) | |
| --- | --- |
| 0 | 10 |
| 2.2 | 0.12 |
| 2.04 | 8.56 |
| 2.16 | 16.44 |
| 7.8 | 1.44 |
| 1 | 17.56 |
| 42.84 | 1.96 |
| 3.48 | 0.36 |
| 6.12 | 2.8 |
| 16.76 | 4.48 |
| 9.44 | 15.68 |
| 8.2 | 0.64 |
| 2.64 | 12 |
| 3.48 | 1.48 |
| 13.44 | 0.12 |
| 11.16 | 0.2 |
| 6.44 | 23.64 |

| OF 2 – High Mobility (s) | |
| --- | --- |
| 0 | 10 |
| 15.72 | 1.52 |
| 3.6 | 9 |
| 1.84 | 12.2 |
| 19.28 | 1.32 |
| 0.8 | 28.16 |
| 19.72 | 5.28 |
| 12.36 | 9.84 |
| 4.84 | 3.36 |
| 26.84 | 7.16 |
| 21.44 | 32.4 |
| 3.92 | 5.24 |
| 8.64 | 3.64 |
| 5.84 | 0.84 |
| 6.36 | 0.72 |
| 13.08 | 1.64 |
| 13.08 | 15.12 |

***Repeated OF – Place Preference***

| OF 1 - Time in Center Zone (s) | |
| --- | --- |
| 0 | 10 |
| 11.20 | 20.36 |
| 31.56 | 123.96 |
| 10.92 | 8.16 |
| 59.32 | 54.28 |
| 16.96 | 94.04 |
| 3.40 | 33.04 |
| 14.40 | 596.36 |
| 3.92 | 28.12 |
| 5.60 | 213.60 |
| 27.92 | 65.68 |
| 77.68 | 30.76 |
| 9.88 | 61.20 |
| 18.80 | 22.20 |
| 12.00 | 42.88 |
| 4.40 | 10.12 |
| 62.64 | 1.80 |

| OF 2 - Time in Center Zone (s) | |
| --- | --- |
| 0 | 10 |
| 31.64 | 30.84 |
| 50.24 | 7.52 |
| 10.88 | 12.52 |
| 28.00 | 17.84 |
| 21.88 | 7.52 |
| 17.00 | 32.32 |
| 28.08 | 17.12 |
| 14.72 | 1.88 |
| 0.48 | 1.64 |
| 9.96 | 1.48 |
| 13.24 | 7.68 |
| 5.48 | 11.40 |
| 19.20 | 58.68 |
| 10.76 | 31.64 |
| 2.04 | 28.88 |
| 69.48 | 0.00 |

| OF 1 - Time in Middle Zone (s) | |
| --- | --- |
| 0 | 10 |
| 37.20 | 51.16 |
| 53.20 | 170.64 |
| 53.64 | 59.32 |
| 63.36 | 30.04 |
| 89.04 | 36.88 |
| 20.48 | 52.64 |
| 51.20 | 3.40 |
| 23.68 | 25.32 |
| 150.00 | 250.96 |
| 84.00 | 101.44 |
| 36.76 | 145.08 |
| 56.16 | 50.60 |
| 83.64 | 43.88 |
| 88.08 | 207.68 |
| 78.40 | 49.36 |
| 190.28 | 9.92 |

| OF 2 - Time in Middle Zone (s) | |
| --- | --- |
| 0 | 10 |
| 179.56 | 96.92 |
| 99.04 | 41.48 |
| 86.32 | 31.72 |
| 161.76 | 61.80 |
| 109.16 | 19.44 |
| 58.24 | 109.92 |
| 166.80 | 34.92 |
| 146.92 | 13.00 |
| 14.80 | 4.24 |
| 39.64 | 10.04 |
| 31.28 | 27.36 |
| 36.88 | 46.04 |
| 100.52 | 102.24 |
| 55.20 | 124.36 |
| 22.44 | 170.72 |
| 206.64 | 1.04 |

| OF 1 - Time in Thigmotaxis Zone (s) | |
| --- | --- |
| 0 | 10 |
| 551.64 | 528.52 |
| 515.28 | 305.44 |
| 534.84 | 532.56 |
| 477.36 | 515.72 |
| 489.52 | 469.12 |
| 576.12 | 514.36 |
| 534.44 | 0.28 |
| 572.44 | 546.52 |
| 444.44 | 135.48 |
| 488.12 | 432.88 |
| 485.60 | 424.20 |
| 534.00 | 488.24 |
| 497.60 | 533.96 |
| 499.96 | 349.48 |
| 517.24 | 540.56 |
| 347.12 | 588.32 |

| OF 2 - Time in Thigmotaxis Zone (s) | |
| --- | --- |
| 0 | 10 |
| 388.84 | 472.28 |
| 450.76 | 551.04 |
| 502.84 | 555.80 |
| 410.28 | 520.40 |
| 469.00 | 573.08 |
| 524.76 | 457.80 |
| 404.92 | 548.00 |
| 438.40 | 585.16 |
| 584.76 | 594.12 |
| 550.44 | 588.48 |
| 555.52 | 565.00 |
| 557.68 | 542.60 |
| 480.32 | 439.12 |
| 534.08 | 444.04 |
| 575.56 | 400.44 |
| 323.88 | 599.00 |

***Ethanol Challenge – Locomotion***

**Control and FG-7142 data are the same as the 0 and 10 µM groups in the OF-NOA tables.*

| OF - Distance Moved (cm) | | | |
| --- | --- | --- | --- |
| Control | FG-7142 | EtOH | FG-7142  + EtOH |
| 7987.30 | 4337.80 | 7637.37 | 6720.38 |
| 6534.13 | 5905.52 | 9012.92 | 2195.73 |
| 5495.34 | 5275.74 | 5452.86 | 6905.72 |
| 8239.49 | 5778.83 | 8022.35 | 2746.65 |
| 4369.52 | 5305.64 | 4631.30 | 7631.90 |
| 8678.79 | 6304.60 | 5841.93 | 4687.80 |
| 4513.12 | 131.85 | 3585.72 | 2651.29 |
| 9846.41 | 6026.64 | 5273.18 | 1429.20 |
| 6563.54 | 8713.96 | 1391.21 | 2893.76 |
| 8024.52 | 7669.88 | 6055.52 | 5325.42 |
| 8727.71 | 1953.75 | 4704.79 | 5418.30 |
| 6262.38 | 9106.96 | 4944.36 | 2910.66 |
| 8144.39 | 6690.43 | 6310.67 | 5526.86 |
| 9643.04 | 8434.40 | 5489.86 | 4342.05 |
| 409.23 |  | 5264.46 | 893.59 |
| 4056.79 |  | 7124.51 | 2946.58 |

| NOA - Distance Moved (cm) | | | |
| --- | --- | --- | --- |
| Control | FG-7142 | EtOH | FG-7142  + EtOH |
| 5705.48 | 2702.76 | 6765.57 | 6352.37 |
| 5671.94 | 1538.85 | 6675.48 | 5170.63 |
| 4204.36 | 655.99 | 4079.00 | 6125.91 |
| 5598.11 | 2877.11 | 5925.47 | 5120.60 |
| 3494.82 | 3398.29 | 3496.57 | 8053.61 |
| 8828.60 | 1402.54 | 4497.44 | 5926.75 |
| 4657.21 | 222.49 | 3704.19 | 6053.96 |
| 7859.24 | 1528.06 | 4735.26 | 3270.57 |
| 4253.52 | 793.85 | 2911.38 | 3198.70 |
| 6734.38 | 895.02 | 6768.06 | 4740.91 |
| 2380.10 | 350.02 | 4294.18 | 5015.93 |
| 4518.49 | 719.13 | 5778.43 | 5710.31 |
| 5681.66 | 4369.75 | 5470.04 | 5186.13 |
| 8404.73 | 611.00 | 5000.43 | 8595.86 |
| 534.75 |  | 3428.42 | 803.75 |
| 1169.10 |  | 5510.75 | 270.18 |

| OF - Immobility (s) | | | |
| --- | --- | --- | --- |
| Control | FG-7142 | EtOH | FG-7142  + EtOH |
| 93.60 | 395.56 | 0.08 | 5.96 |
| 208.04 | 228.12 | 0.08 | 210.08 |
| 289.80 | 230.52 | 12.08 | 38.08 |
| 117.32 | 243.44 | 4.52 | 140.20 |
| 430.44 | 350.20 | 13.56 | 57.36 |
| 112.88 | 231.44 | 1.52 | 125.12 |
| 400.12 | 592.56 | 24.76 | 195.16 |
| 26.72 | 302.92 | 29.12 | 116.60 |
| 261.16 | 80.40 | 135.80 | 67.00 |
| 93.24 | 192.72 | 36.36 | 40.88 |
| 65.32 | 520.00 | 1.48 | 46.64 |
| 149.60 | 89.72 | 10.92 | 85.32 |
| 67.52 | 186.92 | 32.16 | 118.76 |
| 59.68 | 163.64 | 42.36 | 159.32 |
| 582.72 |  | 34.32 | 202.72 |
| 339.96 |  | 2.08 | 111.20 |

| NOA - Immobility (s) | | | |
| --- | --- | --- | --- |
| Control | FG-7142 | EtOH | FG-7142  + EtOH |
| 111.20 | 507.24 | 0.12 | 0.20 |
| 221.52 | 543.40 | 0.20 | 20.84 |
| 359.88 | 579.60 | 4.08 | 0.72 |
| 264.40 | 495.88 | 1.16 | 14.08 |
| 414.36 | 507.36 | 23.08 | 0.60 |
| 48.44 | 553.88 | 0.56 | 0.76 |
| 393.40 | 593.24 | 2.28 | 15.92 |
| 136.20 | 578.32 | 1.80 | 55.92 |
| 475.72 | 565.32 | 5.08 | 68.48 |
| 172.64 | 568.08 | 4.96 | 0.92 |
| 463.44 | 593.36 | 1.96 | 1.64 |
| 348.56 | 586.64 | 1.36 | 1.28 |
| 268.32 | 484.16 | 1.64 | 1.12 |
| 100.44 | 436.76 | 2.72 | 3.40 |
| 576.00 |  | 101.40 | 183.88 |
| 531.40 |  | 1.56 | 119.40 |

| OF - Mobility (s) | | | |
| --- | --- | --- | --- |
| Control | FG-7142 | EtOH | FG-7142  + EtOH |
| 412.48 | 170.4 | 567.84 | 547.6 |
| 356.28 | 360.64 | 535.92 | 357.68 |
| 250.68 | 280.88 | 552.12 | 496.96 |
| 458.16 | 345.84 | 546.04 | 425.36 |
| 105.52 | 188.88 | 527.12 | 494.2 |
| 451.88 | 356.36 | 558.08 | 439.76 |
| 192.76 | 0 | 542.68 | 372.76 |
| 532.2 | 287.12 | 516.36 | 453.16 |
| 327.92 | 507.8 | 432.52 | 511.4 |
| 494.32 | 403.44 | 515.08 | 521.4 |
| 512.12 | 79.16 | 569.96 | 509.96 |
| 433.6 | 499.44 | 561.2 | 484.8 |
| 519.88 | 385.72 | 504.2 | 415.16 |
| 520.16 | 424.08 | 527.44 | 404 |
| 6.72 |  | 498.72 | 376.44 |
| 239.04 |  | 561.2 | 449.88 |

| NOA - Mobility (s) | | | |
| --- | --- | --- | --- |
| Control | FG-7142 | EtOH | FG-7142  + EtOH |
| 261.04 | 62.2 | 568.2 | 565.24 |
| 357.6 | 44.2 | 565.8 | 545.84 |
| 163.16 | 11.84 | 561.96 | 549.56 |
| 325.68 | 96.6 | 567.36 | 555.28 |
| 133.68 | 59.04 | 536.52 | 545.92 |
| 526.84 | 39.64 | 571.32 | 558.76 |
| 201.84 | 0.24 | 567.8 | 538.08 |
| 442.56 | 15.48 | 560.76 | 480.24 |
| 115.88 | 34.32 | 571.72 | 509.04 |
| 419.08 | 29.36 | 562.32 | 565.68 |
| 124.64 | 4.32 | 573.72 | 566.56 |
| 241 | 13.36 | 568.52 | 564.52 |
| 322.44 | 115.56 | 519.84 | 538.84 |
| 482.88 | 17.64 | 576.16 | 537.88 |
| 14.56 |  | 441.04 | 399.76 |
| 58.92 |  | 572.16 | 464.4 |

| OF – High Mobility (s) | | | |
| --- | --- | --- | --- |
| Control | FG-7142 | EtOH | FG-7142  + EtOH |
| 18.28 | 1.08 | 6.56 | 5.88 |
| 29.76 | 4.28 | 41.04 | 7.76 |
| 3.6 | 3.92 | 6.48 | 7.08 |
| 14.76 | 2.52 | 19.44 | 7 |
| 1.72 | 1.68 | 4.16 | 5.8 |
| 28.36 | 6.56 | 6.32 | 8.68 |
| 6.2 | 0 | 6.56 | 5.68 |
| 37.92 | 1.36 | 26.92 | 3.84 |
| 0.76 | 11.8 | 12.4 | 2.08 |
| 2.16 | 3.8 | 28.4 | 6.6 |
| 13.4 | 0.8 | 1.36 | 4.8 |
| 5.64 | 9.12 | 6.52 | 6.04 |
| 4.16 | 7.8 | 2.16 | 1.88 |
| 11.48 | 12.12 | 9.72 | 18.84 |
| 2.92 |  | 23.28 | 4.44 |
| 11.44 |  | 15.08 | 22.52 |

| NOA – High Mobility (s) | | | |
| --- | --- | --- | --- |
| Control | FG-7142 | EtOH | FG-7142  + EtOH |
| 6.04 | 0 | 5.64 | 3.08 |
| 10.64 | 1.48 | 13.2 | 5.68 |
| 2.84 | 0.2 | 5.6 | 0.56 |
| 2.6 | 0.8 | 4.96 | 3.32 |
| 1.96 | 0.04 | 5 | 2.48 |
| 16.4 | 0.6 | 1.36 | 14.92 |
| 4.76 | 0.4 | 3.56 | 4.16 |
| 20.92 | 0.08 | 10.92 | 13.2 |
| 0.68 | 0.2 | 4.16 | 2.16 |
| 0.48 | 2.56 | 13.24 | 2.2 |
| 5.36 | 1.92 | 2.72 | 3.72 |
| 2.88 | 0 | 9.92 | 11.52 |
| 1.4 | 0.24 | 0.72 | 2.56 |
| 8.56 | 2.88 | 4.04 | 38.96 |
| 1.52 |  | 17.44 | 0 |
| 1.76 |  | 6.24 | 0 |

***Ethanol Challenge – Place Preference***

**Control and FG-7142 data are the same as the 0 and 10 µM groups in the OF-NOA tables.*

| OF - Time in Center Zone (s) | | | |
| --- | --- | --- | --- |
| Control | FG-7142 | EtOH | FG-7142  + EtOH |
| 4.20 | 6.64 | 2.92 | 6.96 |
| 8.44 | 31.00 | 3.84 | 36.80 |
| 13.96 | 7.80 | 24.40 | 12.80 |
| 1.28 | 8.00 | 21.12 | 138.20 |
| 5.68 | 6.72 | 15.08 | 74.08 |
| 0.76 | 4.80 | 10.28 | 72.84 |
| 12.00 | 0.00 | 43.84 | 51.44 |
| 6.52 | 5.76 | 56.80 | 26.76 |
| 16.20 | 5.60 | 5.20 | 26.52 |
| 7.24 | 7.88 | 24.72 | 60.00 |
| 0.96 | 6.28 | 39.56 | 28.92 |
| 0.16 | 2.12 | 44.20 | 66.72 |
| 0.88 | 2.76 | 20.36 | 41.92 |
| 1.04 | 2.12 | 62.08 | 174.56 |
| 238.48 |  | 21.68 | 118.32 |
| 6.16 |  | 13.20 | 45.04 |

| NOA - Time in Center Zone (s) | | | |
| --- | --- | --- | --- |
| Control | FG-7142 | EtOH | FG-7142  + EtOH |
| 1.24 | 0.00 | 0.00 | 0.68 |
| 6.92 | 0.00 | 1.84 | 0.20 |
| 19.64 | 1.16 | 1.88 | 3.68 |
| 1.64 | 0.00 | 2.08 | 1.28 |
| 3.76 | 0.60 | 2.64 | 0.88 |
| 0.00 | 1.68 | 10.12 | 0.00 |
| 1.24 | 599.72 | 0.08 | 0.00 |
| 0.84 | 0.12 | 0.16 | 44.04 |
| 0.68 | 304.76 | 0.00 | 0.00 |
| 7.04 | 1.36 | 4.80 | 0.00 |
| 0.00 | 2.20 | 0.00 | 0.68 |
| 1.64 | 2.16 | 0.92 | 0.76 |
| 2.76 | 11.96 | 1.92 | 5.24 |
| 1.88 | 0.00 | 0.00 | 0.84 |
| 0.00 |  | 0.00 | 0.00 |
| 0.00 |  | 1.92 | 100.12 |

| OF - Time in Middle Zone (s) | | | |
| --- | --- | --- | --- |
| Control | FG-7142 | EtOH | FG-7142  + EtOH |
| 14.92 | 58.00 | 18.92 | 36.64 |
| 31.60 | 84.28 | 32.96 | 219.36 |
| 51.72 | 28.80 | 110.28 | 85.68 |
| 12.48 | 37.20 | 53.80 | 55.16 |
| 27.28 | 27.24 | 91.24 | 34.68 |
| 27.60 | 20.52 | 30.60 | 149.56 |
| 122.76 | 599.84 | 163.52 | 181.44 |
| 16.28 | 21.20 | 234.56 | 152.60 |
| 35.32 | 24.68 | 82.36 | 65.88 |
| 19.80 | 30.08 | 60.24 | 72.68 |
| 15.44 | 25.20 | 107.84 | 40.64 |
| 4.68 | 18.12 | 90.96 | 100.52 |
| 10.16 | 21.72 | 51.24 | 134.40 |
| 9.92 | 21.20 | 121.92 | 141.28 |
| 319.24 |  | 77.16 | 99.12 |
| 134.92 |  | 44.68 | 76.88 |

| NOA - Time in Middle Zone (s) | | | |
| --- | --- | --- | --- |
| Control | FG-7142 | EtOH | FG-7142  + EtOH |
| 20.72 | 26.00 | 9.20 | 31.96 |
| 20.64 | 215.16 | 31.84 | 32.20 |
| 65.76 | 92.00 | 99.72 | 78.00 |
| 8.48 | 5.64 | 64.96 | 52.56 |
| 40.04 | 48.00 | 53.52 | 36.32 |
| 0.28 | 90.64 | 58.20 | 12.12 |
| 38.84 | 0.32 | 62.36 | 24.12 |
| 41.08 | 14.28 | 116.40 | 82.80 |
| 117.76 | 44.04 | 2.44 | 6.76 |
| 27.08 | 347.20 | 50.36 | 49.12 |
| 4.04 | 594.16 | 93.12 | 22.20 |
| 40.60 | 197.00 | 43.00 | 101.76 |
| 61.68 | 83.24 | 36.96 | 55.92 |
| 16.84 | 6.92 | 37.48 | 29.04 |
| 485.24 |  | 7.24 | 3.76 |
| 83.76 |  | 93.24 | 4.72 |

| OF - Time in Thigmotaxis Zone (s) | | | |
| --- | --- | --- | --- |
| Control | FG-7142 | EtOH | FG-7142  + EtOH |
| 580.36 | 535.40 | 578.20 | 556.44 |
| 560.00 | 484.76 | 563.24 | 343.88 |
| 534.36 | 563.44 | 465.28 | 501.56 |
| 586.28 | 554.84 | 525.04 | 406.68 |
| 567.08 | 566.08 | 493.64 | 491.28 |
| 571.68 | 574.72 | 559.08 | 377.64 |
| 465.28 | 0.20 | 392.68 | 367.16 |
| 577.24 | 573.08 | 308.64 | 420.68 |
| 548.52 | 569.76 | 512.48 | 507.64 |
| 573.00 | 562.08 | 515.00 | 467.36 |
| 583.64 | 568.56 | 452.64 | 530.44 |
| 595.20 | 579.80 | 464.88 | 432.44 |
| 589.00 | 565.12 | 528.44 | 423.72 |
| 589.08 | 576.72 | 416.04 | 284.20 |
| 42.32 |  | 501.20 | 382.60 |
| 458.96 |  | 542.16 | 478.12 |

| NOA - Time in Thigmotaxis Zone (s) | | | |
| --- | --- | --- | --- |
| Control | FG-7142 | EtOH | FG-7142  + EtOH |
| 578.08 | 574.04 | 590.76 | 567.40 |
| 572.48 | 384.88 | 566.12 | 567.64 |
| 514.64 | 506.88 | 498.12 | 518.36 |
| 589.92 | 594.40 | 533.00 | 546.20 |
| 556.24 | 551.44 | 543.88 | 562.84 |
| 599.76 | 507.72 | 531.72 | 587.92 |
| 559.96 | 0.00 | 532.48 | 575.92 |
| 558.12 | 585.64 | 478.40 | 473.20 |
| 481.60 | 251.24 | 597.60 | 593.28 |
| 565.92 | 251.48 | 544.88 | 550.92 |
| 596.00 | 3.68 | 506.92 | 577.16 |
| 557.80 | 400.88 | 556.12 | 497.44 |
| 535.60 | 504.84 | 561.16 | 538.72 |
| 581.32 | 593.12 | 562.56 | 570.00 |
| 114.80 |  | 592.80 | 596.28 |
| 516.28 |  | 504.88 | 495.20 |

***Opercular Movement Testing***

| Control | | | | | |
| --- | --- | --- | --- | --- | --- |
| Fish | Opercular Beats Per Minute (bpm) | | | | |
|  | 0 min. | 2 min. | 4 min. | 6 min. | 8 min. |
| 1 | 262 | 267 | 269 | 283 | 279 |
| 2 | 280 | 269 | 198 | 252 | 188 |
| 3 | 275 | 267 | 287 | 304 | 344 |
| 4 | 306 | 284 | 254 | 255 | 306 |
| 5 | 262 | 273 | 240 | 310 | 292 |
| 6 | 340 | 322 | 382 | 365 | 368 |
| 7 | 352 | 340 | 304 | 159 | 164 |
| 8 | 327 | 319 | 320 | 254 | 326 |
| 9 | 303 | 277 | 289 | 288 | 287 |
| 10 | 344 | 306 | 289 | 324 | 281 |

| FG-7142 | | | | | |
| --- | --- | --- | --- | --- | --- |
| Fish | Opercular Beats Per Minute (bpm) | | | | |
|  | 0 min. | 2 min. | 4 min. | 6 min. | 8 min. |
| 1 | 288 | 277 | 283 | 285 | 228 |
| 2 | 335 | 334 | 254 | 320 | 246 |
| 3 | 265 | 284 | 309 | 350 | 508 |
| 4 | 352 | 284 | 298 | 271 | 276 |
| 5 | 287 | 298 | 300 | 310 | 309 |
| 6 | 342 | 350 | 359 | 346 | 387 |
| 7 | 331 | 335 | 315 | 258 | 267 |
| 8 | 301 | 292 | 304 | 264 | 297 |
| 9 | 327 | 263 | 264 | 273 | 291 |
| 10 | 276 | 291 | 281 | 283 | 280 |

| EtOH | | | | | |
| --- | --- | --- | --- | --- | --- |
| Fish | Opercular Beats Per Minute (bpm) | | | | |
|  | 0 min. | 2 min. | 4 min. | 6 min. | 8 min. |
| 1 | 240 | 372 | 264 | 303 | 327 |
| 2 | 304 | 304 | 326 | 327 | 342 |
| 3 | 340 | 337 | 346 | 335 | 375 |
| 4 | 331 | 287 | 235 | 310 | 228 |
| 5 | 306 | 304 | 365 | 306 | 382 |
| 6 | 394 | 377 | 283 | 431 | 361 |
| 7 | 329 | 135 | 124 | 134 | 130 |
| 8 | 331 | 315 | 319 | 324 | 294 |
| 9 | 259 | 265 | 287 | 287 | 295 |
| 10 | 315 | 267 | 297 | 284 | 269 |
